# Predicting RNA 3D structure using a coarse-grain helix-centered model

**DOI:** 10.1261/rna.047522.114

**Published:** 2015-06

**Authors:** Peter Kerpedjiev, Christian Höner zu Siederdissen, Ivo L. Hofacker

**Affiliations:** 1 Institute for Theoretical Chemistry, A-1090 Vienna, Austria; 2 Bioinformatics Group, Department of Computer Science, Universität Leipzig, D-04107 Leipzig, Germany; 3 Interdisciplinary Center for Bioinformatics, Universität Leipzig, D-04107 Leipzig, Germany; 4 Research Group Bioinformatics and Computational Biology, University of Vienna, A-1090 Vienna, Austria; 5 Center for non-coding RNA in Technology and Health, Department of Veterinary Clinical and Animal Science, University of Copenhagen, DK-1870 Frederiksberg, Denmark

**Keywords:** RNA tertiary structure, coarse-grain model, knowledge-based energy function, structure prediction

## Abstract

A 3D model of RNA structure can provide information about its function and regulation that is not possible with just the sequence or secondary structure. Current models suffer from low accuracy and long running times and either neglect or presume knowledge of the long-range interactions which stabilize the tertiary structure. Our coarse-grained, helix-based, tertiary structure model operates with only a few degrees of freedom compared with all-atom models while preserving the ability to sample tertiary structures given a secondary structure. It strikes a balance between the precision of an all-atom tertiary structure model and the simplicity and effectiveness of a secondary structure representation. It provides a simplified tool for exploring global arrangements of helices and loops within RNA structures. We provide an example of a novel energy function relying only on the positions of stems and loops. We show that coupling our model to this energy function produces predictions as good as or better than the current state of the art tools. We propose that given the wide range of conformational space that needs to be explored, a coarse-grain approach can explore more conformations in less iterations than an all-atom model coupled to a fine-grain energy function. Finally, we emphasize the overarching theme of providing an ensemble of predicted structures, something which our tool excels at, rather than providing a handful of the lowest energy structures.

## INTRODUCTION

Structured noncoding RNAs (ncRNAs) are an integral part of every cell. In contrast to mRNAs, whose main duty is being the messenger in the construction of proteins from DNA genes, noncoding RNAs are involved in many regulatory and functional processes. In these roles, the three-dimensional structure of an ncRNA is of more importance than the sequence of nucleotides making up the molecule. The structure, however, is largely determined by the self-folding of the sequence.

This structural importance has led to many approaches to predict either the two-dimensional secondary structure (Zuker 2003; Do et al. 2006; Lorenz et al. 2011) or the three-dimensional tertiary structure (Das and Baker 2007; Ding et al. 2008; Parisien and Major 2008; Frellsen et al. 2009; Jonikas et al. 2009; Popenda et al. 2012; Zhao et al. 2012). Compared with the former, predicting the tertiary structure is both costly in terms of computational resources and less accurate than secondary structure prediction. These downsides are, however, balanced by the additional information encoded in the tertiary structure.

In this work, we propose an approach that bridges the gap between abstract secondary structure prediction and concrete all-atomic prediction with a coarse-grained tertiary structure prediction and sampling approach for RNAs. This approach is centered on the helix as the main immutable structural feature.

We provide three interlinked contributions toward predicting RNA 3D structures.

- We first introduce a coarse-grained graph that captures the main structural elements of an RNA structure. It is derived from RNA secondary structures and defines the structural relations of individual helices. Similar graph representations and their use in structure prediction have been mentioned by Zhao et al. (2012), Lamiable et al. (2013), and Kim et al. (2014) but we aim to formalize their definition and illustrate its use as a guide for building a coarse-grain 3D structure.
- Each helix, or consecutive stack of Watson–Crick base pairs in the form of a cylinder, is one coarse-grained building block of our 3D model. Compared with all-atom models, this greatly reduces the number of parameters that need to be considered, while the property of helices of forming regular and consistent structures makes this model feasible. We also give statistics on the actual fit of this cylinder abstraction to observed helices.
- Finally, we provide a sampling algorithm that suggests candidate folded 3D structures, which allows us to explore the ensemble of structures matching a particular knowledge-based distribution of descriptors of the coarse-grain tertiary structure. This leads to a sampling of structures containing not only a realistic local structure but also a plausible global arrangement of the secondary structure elements.

Together these contributions yield a fast algorithm that produces structural predictions competitive with more advanced methods as we will show.

### Other methods and what we contribute

Initial approaches to the prediction of RNA 3D structure simply adapted the methods developed for predicting tertiary protein structure (Das and Baker 2007). This yielded modest accuracy for smaller molecules but suffered from extremely low accuracy for any structure beyond ∼30 nt in length. A subsequent approach broke the structure down into nuclear cyclic motifs which could be assigned energy values and assembled to form full structures (Parisien and Major 2008). The work of Jonikas et al. (2009) introduced a coarse-grained model which focused on the individual nucleotide as the salient building block of the RNA structure and used an energy function based solely on dinucleotide statistics obtained from the corpus of known structures. Such models have been successfully used, e.g., for modeling the folding dynamics of noncoding RNAs (Chen et al. 2013), or characterizing RNA protein interactions (Vincent et al. 2012).

Since the turn of the decade newer approaches have focused on the statistically sound and efficient prediction of local tertiary structure (Frellsen et al. 2009), on the assembly of larger structures based on the knowledge of the structure of existing secondary structure elements (SSEs) (Popenda et al. 2012; Zhao et al. 2012) and motifs (Reinharz et al. 2012). An underlying theme of modern RNA structure prediction approaches is the abstraction of the secondary structure of RNA into distinct elements with distinct properties. With few exceptions, the structure of helices is relatively uniform. Similarly, interior loops, hairpins, junctions and 5′ and 3′ unpaired regions all share certain structural constraints, respectively. In this article, we formalize the definition of each element and introduce a framework for sampling different instances of each element in order to sample the space of coarse-grained 3D structures consistent with the given secondary structure. Whereas previous attempts at reducing the degrees of freedom in an RNA molecule have ranged from using three points to represent a nucleotide (Ding et al. 2008), to using one point to represent a nucleotide (Jonikas et al. 2009), we represent the helix using one line segment and two vectors and consider elements linking helices as the degrees of freedom. It should be noted that a recent approach (Kim et al. 2014) has presented a very similar model using a helix-as-a-stick representation of RNA 3D structure and combining it with predictions of local junction topology to provide accurate predictions of RNA structures. While our approaches overlap in the abstraction of the structure, our method for sampling local structure as well as our energy function formulations differ significantly. Moreover, we emphasize our ability to generate ensembles of structures competitive with the predictions of more sophisticated all-atom models.

The remainder of the article first describes the conversion of a secondary structure to a graph representing the connectivity between the different secondary structure elements. This is followed by a description of the coarse-grain representation of a helix and the methods used to fit a helix to a known all-atom structure. We then shift the focus to the parameters used to assemble tertiary structures and the energy function used to direct the sampling toward realistic structures. We demonstrate the efficacy of this approach in generating structural ensembles that conform to the target distributions and finish with a short comparison to other structure prediction methods. The software implementing this approach is titled Ernwin, is licensed under the GPL-V3 license, and is freely available on Github (http://github.com/pkerpedjiev/ernwin).

## MATERIALS AND METHODS

### Secondary structure elements and graph definition

**FIGURE 1.**
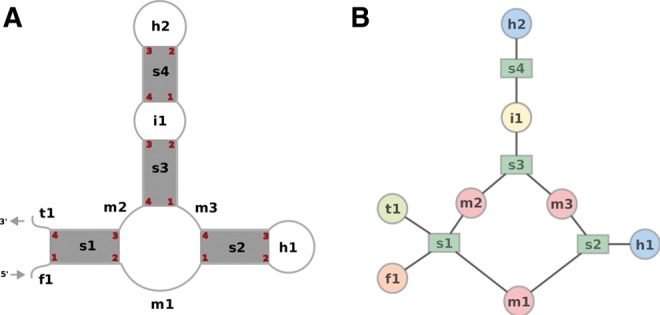
The coarse-grain representation of the 2D structure of an RNA molecule. (*A*) The paired regions are shown as gray rectangles. The arcs show the path of the strand in connecting the paired regions. The labels in black are names given to distinguish the different secondary structure elements in the graph. The elements *f*1 and *t*1 are the 5′ and 3′ unpaired regions, respectively. Elements starting with “s” correspond to base-paired canonical helices. Elements starting with an “h” are hairpins. Interior loops and multiloops are denoted by names starting with “i” and “m,” respectively. The numbers in red indicate the corners of the stem. (*B*) The skeleton graph representation of the structure.

The secondary structure of an RNA molecule can be represented as a collection of elements that share similar characteristics in terms of how they link the canonical helices within the structure. The individual structural elements and their connectivity are depicted in Figure 1. The graph representation (Fig. 1B), which is used to direct the construction of the 3D model, is almost identical to the skeleton graph described by Lamiable et al. (2013), and will be referred to as such in the rest of this article. The following definitions assume the lack of pseudoknots in the secondary structure.

“Stems” are canonical double-stranded helical regions. They are identified by the nucleotides at each “corner,” that is, the nucleotides at the 5′ and 3′ ends of each of the strands (see Fig. 1). The corners are numbered in increasing order from 5′ to 3′ such that *c*_1_(*s*) < *c*_2_(*s*) < *c*_3_(*s*) < *c*_4_(*s*) where *c*_*n*_(*s*) is the index of the nucleotide at corner *n* of stem *s*. Stems may be connected to each other via interior loops or multiloop segments.

The “5′ unpaired region” is the set of unpaired nucleotides at the 5′ end of the molecule. It is defined by the first and last unpaired nucleotides before the first stem. This section is always connected to the first stem. If there are no paired regions, then the entire molecule will be a single 5′ unpaired region.

“Interior loops” are double-stranded regions which link exactly two stems and contain no canonical base pairs, although they may be rich in noncanonical base pairs (Leontis et al. 2006). These regions always connect corners 2 and 3 of one stem (*s*_*j*_) to corners 1 and 4 of the next stem (*s*_*k*_), where the term “next stem” implies that *c*_1_(*s*_*j*_) < *c*_1_(*s*_*k*_). Since no pseudoknots are allowed in our representation we have *c*_2_(*s*_*j*_) < *c*_1_(*s*_*k*_) < *c*_4_(*s*_*k*_) < *c*_3_(*s*_*j*_). Interior loops are defined by the nucleotides *c*_2_(*s*_*j*_) + 1, *c*_1_(*s*_*k*_) − 1, *c*_4_(*s*_*k*_) + 1, and *c*_3_(*s*_*j*_) − 1 for *s*_*j*_ and *s*_*k*_ next to each other. If one strand of an interior loop has no unpaired bases, then the interior loop is defined only by the unpaired nucleotides on the other strand. The interior loop *i*_1_ in Figure 1 connects the two stems *s*_3_ and *s*_4_.

“Multiloop segments” are single-stranded unpaired regions which connect two stems that are not separated by an interior loop. They can connect two stems *s*_*j*_ and *s*_*k*_ where *s*_*j*_ < *s*_*k*_ in three different ways: *c*_2_(*s*_*j*_) → *c*_1_(*s*_*k*_), *c*_4_(*s*_*j*_) → *c*_1_(*s*_*k*_), and *c*_3_(s_*j*_) → *c*_4_(*s*_*k*_). In Figure 1 there are three multiloop segments: *m*_1_, *m*_2_, and *m*_3_.

The “3′ unpaired region” denotes the unpaired nucleotides at the 3′ end of the molecule. This region only connects with the last stem in the structure (*s*_*l*_) and is defined by the nucleotide *c*_4_(*s*_*l*_) + 1 up to the final 3′ nucleotide.

#### Creation of the secondary structure graph

The secondary structure graph is created from RNA secondary structure predictions. Currently, we use RNAfold from the ViennaRNA v2 package (Lorenz et al. 2011). The coarse-grained graph can be trivially created from any secondary structure representation or prediction algorithm (i.e., minimum-free energy folding, centroid structures, nonphysics based methods) which does not contain pseudoknots. Threading a coarse-grain model onto a known 3D structure requires the extraction of the secondary structure, for which we use the annotation produced by MC-Annotate (Gendron et al. 2001), removing the pseudoknots (conflict elimination method) (Smit et al. 2008), creating the secondary structure graph and then fitting helices onto the all-atom model to get the 3D coordinates of the coarse-grain representation (see next section).

### The helix and the 3D model

**FIGURE 2.**
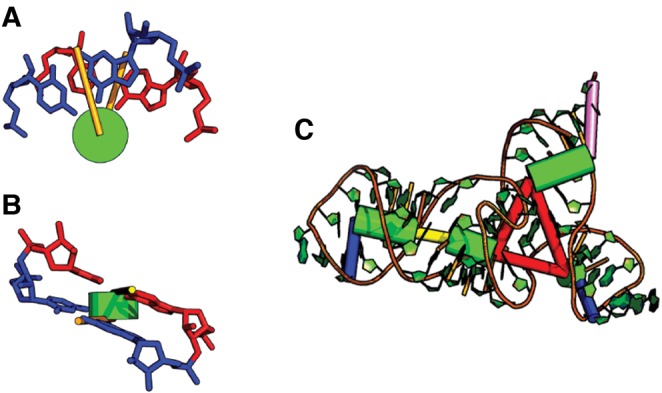
An illustration of the helix model for the 53 nucleotide SMK box (SAM-III) Riboswitch RNA structure (*C*, PDB: 3e5c). The helices are shown as green cylinders, interior loops as thinner yellow cylinders, multiloops as collections of red cylinders, hairpin loops as thin blue cylinders, and the 3′ unpaired region is shown in magenta. A 5′ unpaired region is missing from this structure since the first nucleotide is already paired. We denote the twist parameters as orange lines protruding from the axis of a helix as viewed along (*A*) and perpendicular (*B*) to the axis of a helix. Each one is perpendicular to the cylinder axis and points in the direction of the midpoint between the C1′ atoms of the first and last base pair in the helix. In *C*, twist vectors are interpolated for the base pairs in the middle of the stem with values stored only for the vectors at the end of each stem.

At the core of the Ernwin tertiary structure prediction package is the reduced cylinder-like model of an RNA helix. The representation of the helix is defined by a line segment indicating the start and end points of the axis of the helix (*a*_*s*_, *a*_*e*_) as well as two vectors pointing from the ends of the axis to the middle of the first and last base pairs, respectively (*t*_*s*_, *t*_*e*_) as depicted in the schematic (Fig. 2; Supplemental Fig. A.10). The calculation of these parameters cannot exactly represent a helix insofar as RNA helices deviate from an ideal double helix. While such a representation has previously been alluded to (Laederach et al. 2007; Popenda et al. 2012), the calculation of the axis and twist vectors has never been explicitly defined. We tested four different methods for fitting idealized helices to real RNA double helices, the details of which are documented in Supplemental Section A.5. The position of the twist values is illustrated in Supplemental Section A.5.5 and Supplemental Fig. A.10.

### Proposal distribution, model building, and sampling

The proposal distribution for new structures is based on a set of statistics relating the orientation of two adjacent helices, the orientations of hairpin loops, and the 5′ and 3′ unpaired regions relative to helices. Just as the position of 1 nt relative to the previous can be expressed as a function of the torsion angles and sugar pucker, the position of one coarse-grain helix relative to the previous can be expressed using a set of six different parameters (subsequently referred to as interhelical parameters) (Bailor et al. 2011; Sim and Levitt 2011). Likewise, much as the distribution of potential torsion angles can be inferred by looking at solved structures, so can the range of potential interhelical parameters. This distribution, which should exclude parameters which lead to impossible configurations (due to steric hindrances, for example), is partitioned according to the size of the secondary structure element (i.e., interior loop or multiloop), which separates the two stems (see “Secondary structure elements and graph definition”).

The current implementation uses parameters mined from a large corpus of predicted 3D structures to ensure there is no overlap between the tested structures and the statistics used to predict them. This approach can be used to supplement statistics mined from known structures in cases where no instances of a particular secondary structure element are known. Each predicted 3D structure was created from a random sequence whose secondary structure was predicted using RNAfold (Lorenz et al. 2011) and whose 3D structure was predicted using FARNA (Das and Baker 2007).

The 3D model is initially built by sampling orientation parameters for every interior loop, multiloop, hairpin loop, 5′ and 3′ unpaired region. Using the parameters for a coarse-grain element is analogous to inserting a fragment for that element into the overall structure. The length and twist parameters for each stem are also sampled from the list of known parameters to account for the slight variability seen in the structure of canonical helices. The initial model is built by traversing the skeleton graph (Fig. 1B) and placing each element in relation to the one preceding it. Due to the cyclical nature of junctions, one segment is necessarily determined by the orientations of the other segments. A break is introduced in the multiloop segment with the largest number of nucleotides of all the segments in a particular junction.

Direct sampling from the proposal distribution produces structures that have native-like local structure but lack long-range tertiary interactions and global structural properties found in real structures. We therefore need to add an energy function that enforces global features, such as compactness of structures, and favorable long-range interactions, which we will describe below. In order to sample from the corresponding distribution we implement a Markov chain Monte Carlo (MCMC) simulation. At each sampling step, one loop or stem is picked at random, its parameters are resampled, and the resulting structure's energy is evaluated. The new structure is accepted or rejected according to the Metropolis Hastings rule using the energy function. The secondary structure is kept fixed during the entire simulation.

### Energy function

Before explaining the energy function, we will state the definitions of a few commonly used terms:

Measure: some quantifiable property of a 3D structure (e.g., its radius of gyration).
Proposal distribution: the distribution of structures obtained using only the statistics on the orientation of adjacent helices.
Target distribution: the desired distribution of a measure, i.e., the distribution observed in native structures of the appropriate size (i.e., smaller structures will have a greater chance of having a lower radius of gyration).
Sampled distribution: the distribution of a measure among all of the structures sampled over the course of a simulation.
Background distribution: the sampled distribution of a measure for a simulation run using only the constraint energies, equivalent to the distribution of the measure induced by the proposal distribution.
Reference distribution: the distribution used to calculate the energy values by comparison to the target distribution. Initially derived from a set of decoy structures (see below), the reference distribution approaches the sampled distribution as more samples are added from the MC simulation.

Our energy function is composed of five separate terms each of which is described in one of the next subsections. Two are based on physical forces to exclude impossible structures (called constraint energies, and described in the subsections “Clash detection” and “Junction closure detection”), the remaining three are knowledge-based potentials derived from known structures (called nonconstraint energies, and described in Radius of gyration, A-minor energy, and Loop–loop interaction energy). For comparison we also use an energy function which returns a value of zero for every structure (leading to constant acceptance of new structures and a direct sampling from the proposal distribution) and is intended to mimic the effect of using no energy.

The knowledge-based potentials are based on coarse-grained measures whose distributions differ between native structures (target distribution) and structures sampled from the proposal distribution (reference distribution). For each of these coarse-grained measures, we will present examples of the target distribution and the reference distribution (as calculated from a decoy) as well as the associated energy calculated by the reference ratio method (Hamelryck et al. 2010; Valentin et al. 2014). The energy associated with a value *x* of the measure is calculated as the log of the ratio of the target distribution [*p*_*t*_(*x*)] divided by the reference distribution [*p*_*r*_(*x*)] and multiplied by a factor *c* which serves as a parameter for tuning how closely the target distribution should match the sampled values (see Supplemental Section A.6.2):

$$E=-c^{*}\log\frac{\;p_{t}(x)}{\;p_{r}(x)}$$

The target distribution is defined by subgraphs of the ribosome structure (PDB: 1JJ2). For a given structure we calculate the measure of interest on all subgraphs whose sequence length lies within a certain range of the target structure. The range is initially very narrow (within 1% of the length of the target structure) but is expanded until there are at least 500 measures that can be used to define a probability distribution for the target measure. For example, if trying to model a structure with a length of 100, we would consider the radii of gyration of all ribosomal subgraphs with a length between 100 − *x*, and 100 + *x*, such that the number of available subgraphs within that range is >500.

The background, or reference distribution, is initially approximated from random subgraphs of an artificial ribosome structure (decoy) built using only the proposal distribution and the constraint energy terms. In a typical knowledge-based energy function, this corresponds to the reference state (Sippl 1995) and remains unchanged throughout the simulation. As pointed out in Hamelryck et al. (2010) and Valentin et al. (2014), however, the reference state depends on the structure being sampled. The reference state for the molecule being simulated is initially unknown, but can be approximated over the course of the simulation. This leads to a reference distribution which changes to reflect the ensemble of sampled structures. The energy function is therefore variable at least during the burn-in phase of the simulation.

As more samples are produced by the MCMC, they are added to the reference distribution and used in the calculation of subsequent energies. Gaussian kernel density estimates are used to convert discrete frequencies into continuous distributions for both the target and sample distributions using a bandwidth selected using Scott's rule (Scott 2009). The bandwidth selection for the kernel density estimates smooths the distributions obtained from the training data relieving the threat of trying to match a distribution specific to the substructures of the ribosome which were used to estimate the parameters of the energy function. Ernwin recalculates the reference distribution after every tenth MCMC step. This leads to a convergence of the distribution of sampled coarse-grain measures to their target distribution. It should be noted that while the reference ratio method (Hamelryck et al. 2010; Valentin et al. 2014) uses multiple complete sampling runs (iterations) to adequately describe the reference distribution such that samples are drawn from the target, we re-create it multiple times over a single simulation, thus enabling a close approximation of the target distribution over some predictable burn-in period (see “Energy function quality and simulation length”).

**FIGURE 3.**
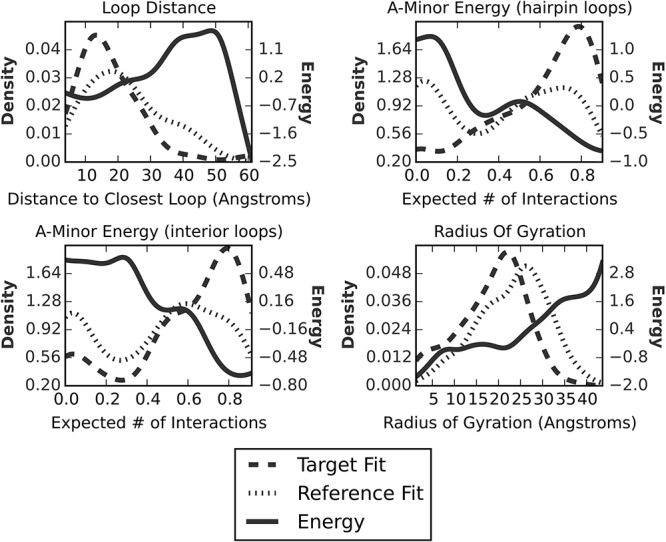
Frequency distribution and corresponding initial potential for the four different energy terms (Loop–loop distance [cf. subsection “Loop–loop interaction energy”], radius of gyration [cf. subsection “Radius of gyration”], and A-minor energy for interior and hairpin loops [cf. subsection “A-minor energy”]). The target (dashed) and reference (dotted) distributions are obtained from subgraphs of the native ribosome and a decoy ribosome structure obtained by simulating using only constraint energies (i.e., clash detection and junction closure), respectively. Here we used subgraphs of length 83, the length of the *Escherichia coli* thi-box riboswitch 2HOJ.

An illustration of the calculation of each of the nonconstraint energy functions is shown in Figure 3. Immediately visible is the tendency for the energy to decrease in the regions where the probability density of the target distribution is greater than the probability density of the reference distribution. In practice, the reference distribution and the concomitant energy function change according to the values of the structures sampled over the course of the simulation. This process is described in more detail in Supplemental Section A.6.1.

#### Clash detection

To prevent two or more atoms from occupying the same space, a heavy energetic penalty is imposed in such situations. As our model does not track individual atoms, such an energy function has to be somewhat indirect and imprecise. We have little to no hope of detecting clashes between nucleotides which are not part of a helix. There is simply too much variation in their spatial position, given the parameters that define our model. The position of the remaining nucleotides, which are in helices, can reasonably be approximated and accounted for (see Supplemental Section A.1.1). These estimated positions will be referred to later as the virtual base pair and virtual atom positions. Any clashes within the atoms of these nucleotides are given a heavy energetic penalty to ensure the rejection of that conformation.

#### Junction closure detection

The construction of multiloops by placing subsequent helices independently one after another leads to the problem that the parameters of the final segment of a multiloop will necessarily be determined by the previously sampled segments. Since this set of parameters is calculated, rather than chosen from the known values, it is possible that it corresponds to a sterically impossible structure, e.g., when the distance between the ends of the two adjacent stems is too large to be bridged by the nucleotides in between. To counter this occurrence, we penalize such situations by imposing a large energetic penalty. The allowed distances are determined as a function of the distance between the positions of the virtual P and O3′ atoms of the capping nucleotides of the two adjacent stems.

#### Radius of gyration

Like proteins, albeit in a less pronounced manner, RNA molecules tend to form compact structures. To measure the compactness of the structure, we use the common radius of gyration (ROG) measure as calculated over the virtual residues of the stems of the structure (see “Clash Energy,” Supplemental Section A.1.1). Instead of simply giving a bonus for a more tightly packed structure, we aim to sample structures whose distribution of ROG values matches the distribution we would expect from typical structures of that size.

#### A-minor energy

The A-minor motif is the most common long-range interaction found in RNA structures and contributes greatly to the overall tertiary fold of the molecule (Nissen et al. 2001). It involves an interaction between an unpaired adenine with the minor groove of a helix. The unpaired adenine (the donor) may be found in hairpins, interior loops, or junctions, but only instances where it occurs in a hairpin or interior loop are considered in this paper. Predicting the positions in the secondary structure where such an interaction might occur is difficult. We therefore assign a probability of forming an A-minor interaction to each helix–loop pair and score each loop by the weighted number of its A-minor interactions.

**FIGURE 4.**
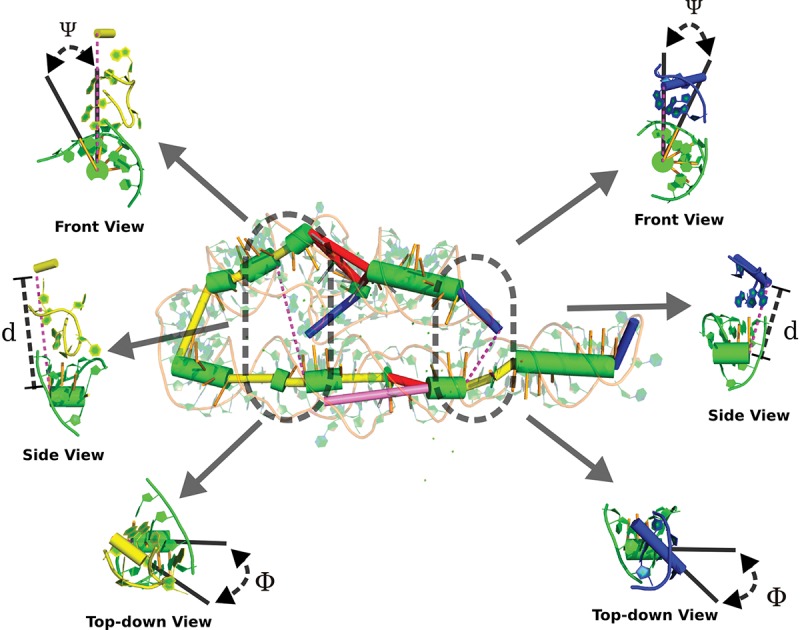
The parameterization of A-minor interactions in the Group I intron (PDB ID: 1GID). On the *left* is an interaction between an interior loop and a stem, while on the *right* is an interaction between a hairpin and a stem. Both are parameterized in the same manner, wherein the distance (*d*) along the interaction vector (the vector between the two closest points on the two interacting elements) is shown in the *side* view, the angle between the interaction vector and the minor groove of the stem (ψ) is shown in the *front* view, and the angle between the two interacting elements (φ) is shown in the *top-down* view. The direction of the view is relative to the receptor stem.

If we imagine that the interaction between a helix and a loop occurs over a vector connecting the closest points of the two elements, then we can parameterize it using its distance *d*, the angle it makes with the minor groove of the stem (ψ) and the angle (ϕ) between the axes of the two elements, as depicted in Figure 4.

We now estimate the probability distribution for true A-minor interactions *P* (*d*, ϕ, ψ|*I*) as well as all helix–loop pairs *P* (*d*, φ, ψ) from the native ribosome structure. We can then calculate the probability that two elements (*i*, *j*) interact given their relative positions *d*, ϕ, and ψ:

$$P_{i,j}(I|d,\varphi ,\psi )=\frac{P_{i,j}(d,\varphi ,\psi |I)\times P_{i,j}(I)}{P_{i,j}(d,\varphi ,\psi )}.$$

**FIGURE 5.**
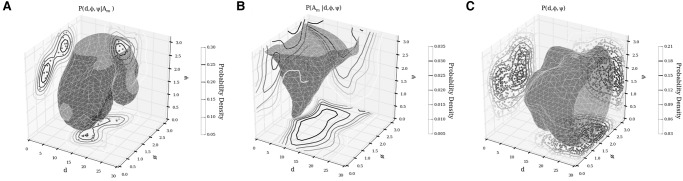
Cross sections and iso-surface showing the probability density of the parameters describing hairpin to stem A-minor interactions. (*A*) The probability density of seeing interaction parameters (*d*, φ, ψ) given an A-minor interaction. (*B*) The probability density of an interaction as calculated using Bayes’ law. (*C*) The probability density of seeing a particular set of parameters among all adenine-containing hairpin loops within 30 Å of each other. The iso-surface in each plot corresponds to the mean probability density of all the points on the 3D grid describing the parameter space. The same plots for interior loops are presented in Supplemental Figure A.1.

Figure 5 shows the probability distributions introduced above for hairpins. As expected, elements engaged in hairpin A-minor interactions are closer to each other and their interaction vector is generally more anti-parallel to the receptor minor groove than in the general population of pairs of proximate elements. The angle between the donor and receptor elements (ϕ) varies less, but shows a split toward a bimodal distribution in the interacting population. The distributions of parameters for interior loop A-minor interactions are similar although the minor groove–interaction angle (ψ) varies slightly more; see Supplemental Figure A.1. This is likely explained by the tendency for the A-minor interactions to occur at locations that do not correspond to the closest point between the coarse-grain interior loop donor and its stem receptor (see Supplemental Fig. A.2).

To obtain an energy function we calculate the expected number of A-minor interactions *A*_*i*_ that a particular loop, *i*, is involved in, by summing over all possible interacting helices which are not directly connected to loop *i*. Elements further than 30 Å have an almost negligible probability of participating in A-minor interactions and are therefore excluded.

$$A_{i}(I|d,\varphi ,\psi )\sum_{\;j\;\in \;A,\mathrm{dist}(i,j)\;\leq \;30}P_{i,j}(I|d,\varphi ,\psi ).$$

**FIGURE 6.**
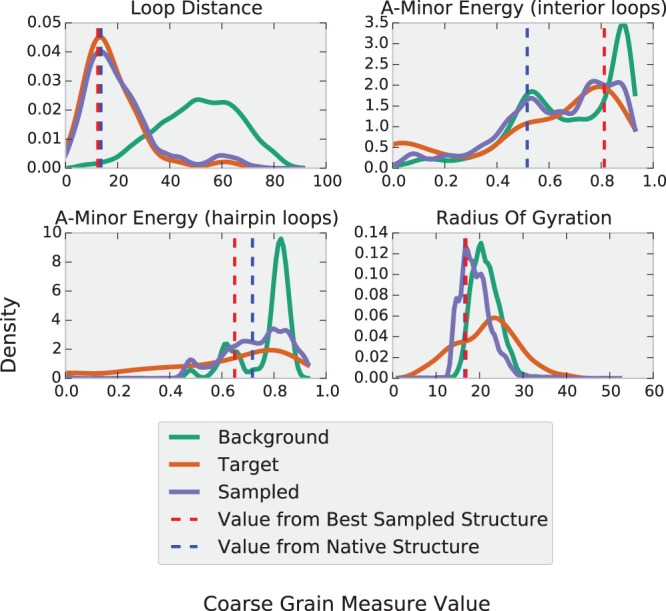
The four different coarse-grain measures and their distributions as applied to the *E. coli* thi-box riboswitch (PDB ID: 2HOJ, length: 83 nt). After including an energy value for the coarse-grain measures, the structures sampled begin to adopt values (“sampled” distribution *above*) similar to those expected from native structures (“target” distribution *above*). The radius of gyration is only slightly affected due to the constraints imposed by the topology of the RNA molecule. The blue and red dashed lines show the measures as calculated for the native and best sampled structure. The graphs for the loop distance, A-minor (interior loops), and A-minor (hairpin loops) are presented for the first hairpin, the first interior loop, and the first hairpin, respectively. A separate energy term is created for each element that the energy applies to.

Like all other energy terms, we obtain a target distribution from the ribosome and use the log odds ratio (Equation 1) to assign an A-minor energy to each loop. The corresponding distributions and energy function can be seen in Figure 6. As expected, the distribution for the native ribosome structure (target distribution) is shifted toward higher number of A-minor interactions compared with the reference distribution obtained from a decoy.

#### Loop–loop interaction energy

Unlike proteins, RNAs are polar molecules and thus lack the innate tendency to form tightly clustered structures. Their packing is more reliant on the presence of interacting motifs which tend to attract each other (Butcher and Pyle 2011). Among the variety of interactions which stabilize the global tertiary fold of an RNA molecule is the hairpin–hairpin interaction. This often occurs when two proximate hairpins are linked via hydrogen bonds and/or base stacking interactions. While there are attempts to predict such interactions (Theis et al. 2010; Sperschneider et al. 2011), we do not presume to have this ability and instead try to sample structures which have native-like distances between the hairpins. The ribosome provides a training set from which to observe a distribution of distances from one hairpin to its nearest neighbor. This distribution, along with its analog from the background distribution of the thi-box RNA are shown in the upper left plot of Figure 6. In this structure, the loops happen to interact, but in cases where they do not it is expected that this energy will be balanced by potential A-minor interactions elsewhere or by the constraints of the local tertiary structure. An instance of this energy is created for each hairpin in the structure.

## RESULTS

### Structure sampling

Coarse-graining RNA structure to the level of secondary structure elements provides a fast, logical way of sampling only the regions whose 3D structure varies the most. We sampled using a Markov Chain Monte Carlo simulation for 10,000 iterations. Every nonclash structure was stored and used to calculate summary statistics about the distributions of the coarse-grain variables. In the Supplemental Material we show that as the simulation progresses the deviation between the target and sampled distributions decreases, indicating the efficacy of our sampling approach and hinting toward a potential criterion for when to terminate the simulation (see Supplemental Section A.6.2). The results indicate that applying energy functions which take only these coarse-grain elements into account can shift the distribution of sampled structures toward the native. To compute the similarity between two structures, we use the commonly used root mean square deviation (RMSD) between the superimposed positions of the virtual residues (see “Clash Energy,” Supplemental Section A.1.1) of their coarse-grain helix model representations (Kabsch 1976). The resulting structures are comparable in RMSD to the ones created by other tools such as FARNA (Das and Baker 2007) and RNAcomposer (Popenda et al. 2012).

By applying the described energy functions, the sampling can be directed toward regions of the conformation space that share similar characteristics with native structures. In the case of the radius of gyration, constant-energy sampling yields larger, more spread out structures due to the preference for coaxial arrangements of helices (see Supplemental Fig. A.3).

Figure 6, which uses the *E. coli* thi-box riboswitch as an example, shows that structures sampled with no energy function tend to have a radius of gyration of slightly >20 Å. Structures sampled using an energy function including a term for the radius of gyration have a radius of gyration distribution peaking at ∼18 Å. The application of the energy term has slightly broadened the distribution of sampled structures, which fortuitously happens to peak at the true value of ∼18 Å. Clearly visible in this example is the limitation in trying to sample from the target distribution. As it includes structures smaller and greater than the native, it is more spread out and cannot be adequately approximated by the topology of the thi-box riboswitch structure. Fortunately, for larger structures, such effects become less noticeable due to the greater variety of conformations that can be adopted by larger structures.

The other two energy terms exhibit a pattern more in line with our expectations than that of the ROG energy. The A-minor energy for interior and hairpin loops (Fig. 6, upper right and lower left, respectively, for the thi-box riboswitch [PDB ID: 2HOJ] and Supplemental Figures A.7, A.8 for all other structures) is broadened to resemble the target distribution. The peaks of the distribution, while slightly displaced from the native values are shifted toward them as compared with the background distribution.

The loop distance values for structures sampled with the constraint energy function are centered around a distance of 50 Å, while the real value is closer to 10 Å (Fig. 6, upper left). This is due to the presence of a kissing hairpin interaction in the structure and is reflected in both the target and sampled distributions. It should be noted that the target distribution includes structures which have hairpins that do not interact and thus peaks at a value beyond that expected for a structure with interacting hairpins. Nevertheless, the distribution of sampled structures is shifted in the direction of closer hairpins.

**FIGURE 7.**
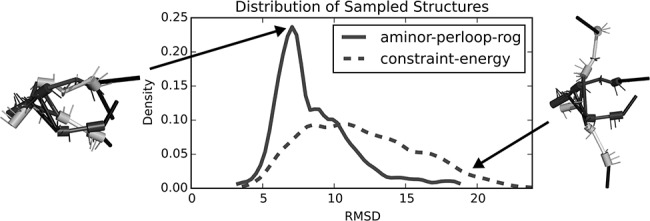
A visualization of the best (lowest energy) structure for the *E. coli* thi-box riboswitch (2HOJ, see “Prediction quality”) sampled using using the full energy (*left*) versus the constraint energy (*right*). The darkened structure is native whereas the lighter is sampled. The plot shows the shift toward sampling more native-like structures using the full energy as opposed to the constraint energy. Superpositions of the lowest energy models and the native structures are provided for the whole benchmark set in Supplemental Table A.1.

A stark example of applying the described energy functions to the sampling of a particular structure is illustrated in Figure 7. The distribution of RMSD values of the structures sampled with a constant-energy function (“constant-energy”) has a weighted mean at a value of ∼13 Å. Applying our energy function shifts the weighted mean of the RMSD distribution to a value slightly >7 Å. By visualizing the lowest energy sampled structure, we see a marked qualitative improvement in the model from the energy-based sampling as compared with the constraint energy sampling (Fig. 7, top left and top right, respectively). The shift toward lower RMSD does not always occur, as for the example of 3R4F (see Supplemental Section A.4), but in the RNAs tested, the general trend was toward an improvement. The results for all of the tested structures are shown in Supplemental Figure A.4.

Supplemental Figure A.6 shows the target, background and sampled distributions for a number of solved structures.

### Comparison with other structure prediction methods

#### Prediction quality

**FIGURE 8.**
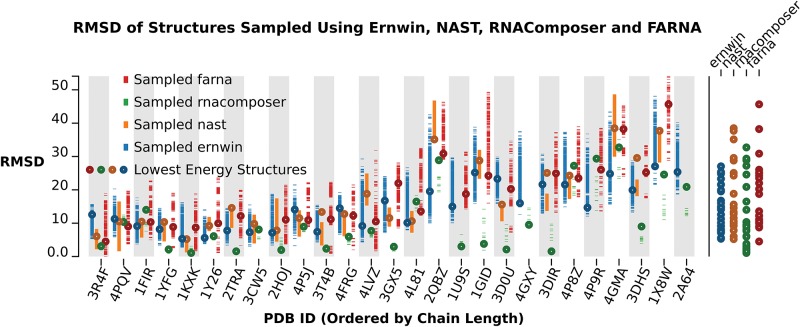
A comparison of the RMSD value between the structures sampled by each program and the native structure. Each dash in the chart represents one sampled structure. The circles represent the lowest energy structures. On the *right* is a tabulation of the lowest energy structures predicted by each program. The RMSD values were calculated by threading a coarse-grain representation onto the all-atom models generated by the other programs. Missing values indicate that the corresponding program failed to give a prediction for that structure.

The overall quality of sampled structures is comparable to some of the best structure prediction programs available. By calculating a coarse-grain model from the structures predicted using FARNA and RNAcomposer (where we provide the true secondary structure) we provide a comparison of the alignment between the predicted and native structures using the RMSD metric (Fig. 8). The structures used for the benchmarks were collected from the BGSU RNA 3D Hub nonredundant RNA structures list (Leontis and Zirbel 2012) and filtered to exclude structures with <70 or >500 nt as well as multimers and RNAs with bound proteins.

**FIGURE 9.**
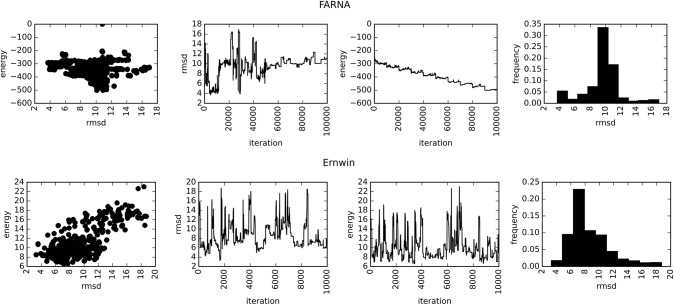
Statistics for the structure prediction procedure of FARNA (*top*) and Ernwin (*bottom*). The energy of the best structure constantly decreases up to 1,000,000 iterations with FARNA, whereas it plateaus very rapidly with Ernwin. This indicates that the broad conformational space has been mostly explored shortly within the start of the Ernwin simulation and subsequent MC steps only explore around the low-energy basin. The *left* plots display the RMSD of the structures as a function of their energy, where Ernwin displays the desired correlation. The next plot shows the RMSD of the structure as a function of the MC iteration showing no clear downward trend as the simulation progresses. The third plot shows the energy as a function of the iteration number showing the clear downward trend throughout the entire FARNA simulation and the quick arrival at a steady for Ernwin. Finally the histogram shows the RMSD of every structure sampled by both methods. The RMSDs are not directly comparable as FARNA's are for an all-atom model while Ernwin's are calculated over the coarse-grain representation.

An example of a relatively successful simulation using Ernwin is shown in Figure 7. The conformation of the lowest energy structure has two helical arms arranged in a roughly parallel fashion with the two hairpin loops near to each other, whereas a random structure sampled using a constant energy shows a worse configuration where each of the arms of the structure points in a separate direction and the loops are on opposite ends of the molecule. The quality of the better prediction is largely due to the accurate sampling of most coarse-grain measures shown in Figure 6. The loop distance, expected number of hairpin loop A-minor interactions, and the radius of gyration are sampled at values extremely close to the native. The sampled values for the entire ensemble match the target values very well, except for the case of the ROG which is, in this case, constrained by the topology of the secondary structure. An examination of the energy landscape (Fig. 9) indicates that our energy function describes this structure particularly well, showing the desired negative correlation between RMSD and energy. While this is the case for most structures (Supplemental Table A.2), there are some notable exceptions that lead to poor predictions. One of these is presented and discussed in more detail in Supplemental Section A.4.

The circled RMSD values in Figure 8 correspond to the lowest energy structure for FARNA, Ernwin, and NAST. For RNAcomposer they correspond to the structure returned when asking for one structure. As is expected, smaller structures are predicted with greater accuracy than larger ones. RNAcomposer performs exceptionally well on a handful and significantly worse on others. This is likely explained by its use of known fragments for the interior and multiloop sections, leading to near exact matches for structures with unique junction topology and sequences (i.e., tRNA, 2TRA). FARNA exhibits more tempered performance over the smaller structures which degrades over the larger structures and Ernwin exhibits measured performance over the whole data set. Over the entire sampling run, Ernwin consistently and thoroughly samples a wide range of available conformations, often yielding structures in the more native range of the samples. FARNA can sample a wide range of values, but does so more sparsely which is likely due to the fact that its simulated annealing approach falls into an energy basin that is difficult to escape as the temperature decreases. This performs well in the context of smaller structures, but leads to poor sampling of larger structures. In such cases, Ernwin can sample more structures closer to the native than both FARNA and RNAComposer.

NAST samples many structures but in very narrow ranges of the conformational landscape whereas RNAComposer only returns a maximum of 10 structures. The seemingly exemplary performance of RNAComposer in sampling low-RMSD structures should be looked upon with slight suspicion due to its use of loop topology and sequence to pick out large fragments for constructing sampled structures. Given the presence of the benchmark structures in the PDB database, these fragments are likely in RNAComposer's database of building blocks and thus accurately assembled into the known structures. While this works well with structures containing seen-before and unambiguous motifs, it can quickly backfire when a motif is absent from the database, or has multiple geometries (as may be the case for the structures 4GMA or 4P9R).

A thorough and wide sampling of the potential solution structures, as provided by Ernwin, can help explore the enormous conformational space accessible to larger RNA molecules and provide many potential structures for further examination. It quickly samples unique structures (see Supplemental Table A.4 for timing information), and can be readily expanded with more accurate and more numerous fragments to expand the range of accessible conformations.

#### Energy function quality and simulation length

One of the challenges in structure prediction is determining the burn-in period before sampling structures from the distribution, as well as determining the thinning factor. This, of course, depends on the sampling procedure as well as the energy function. In an attempt to quantify this, we recorded the energy value and RMSD of the current structure at each iteration of the simulation (see Fig. 9). In contrast to FARNA, Ernwin quickly reaches a locally minimal energy and samples structures around it. The histogram of RMSD values indicates the propensity for sampling low-energy, low-RMSD structures. FARNA, in contrast, continuously finds lower energy structures throughout the entire simulation, but due to the lack of correlation between the energy and RMSD (left panel) ends up sampling many suboptimal structures.

While FARNA's energy function works well for smaller structures, it seems to fail for larger structures and leads to sampling of high-RMSD conformations. Ernwin's energy function, based on global helical arrangements provides a more robust measure of the general quality of a structure. While the example provided in Figure 9 is particularly fortuitous, most structures tested show a characteristic linear correlation between the energy and RMSD of the sampled conformations (see Supplemental Table A.2 for Ernwin and Supplemental Table A.3 for FARNA). By examining the trajectory of the sampled energy values, we propose that Ernwin achieves an adequate sampling within <2000 iterations, whereas FARNA requires many more iterations to reach the lowest energy values. Given Ernwin's method of sampling coarse-grain measures from target distributions, one can also assess how well it has sampled from each distribution by examining the Jensen-Shannon divergence (Endres and Schindelin 2003) of the sampled values from the target values (see Supplemental Section A.6.2 and Supplemental Fig. A.13). When the divergence levels off, we have adequately sampled from our target distribution indicating that additional MC steps provide no new conformations. While there is no consistent number of iterations that is applicable to all structures, examining the progress of the distribution can provide an empirical method for determining when to end a simulation. A thorough treatment of this topic, however, is out of the scope of this paper and is merely mentioned to highlight the utility of having a probability-distribution based energy function.

## DISCUSSION AND CONCLUSION

In this paper, we have presented a coarse-grained model of RNA structure parameterized by the angles and shifts between helices. We have shown that coupling a simple proposal distribution with a probability-based energy function can yield predictions that match those of programs with much more sophisticated models and energy functions. We propose that our model can be used for quick exploration of the macroscale conformational space of an RNA molecule.

We suggest that such a model can also be useful for the elucidation and identification of different RNA species in atomic-force microscopy images where the positions of the individual atoms are largely indistinguishable (Petkovic et al. 2015). Given fluorescence resonance energy transfer (FRET) data, the structures generated by our model can provide the experimentalist with an overview of the global structure of the RNA molecule without the overwhelming precision (and uncertainty) of an all-atom model. Simple diagnostics such as determining whether two loops have the potential to localize within a certain distance of each other, while maintaining steric integrity, can also easily be performed.

A particularly compelling future application is the combination of our sampling method with data from a SAXS experiment. As RNA in solution can adopt a multitude of conformations, its true structure in a solution may not be accurately represented by the crystal structures used as benchmarks (Ali et al. 2010; Brenner et al. 2010). Spectra obtained from SAXS experiments, however, reflect the true distribution of conformations present in a solution. Furthermore, coarse-grained models as presented here, are sufficient to generate theoretical SAXS profiles. Thus, SAXS data could be incorporated directly in the simulation as an additional potential based on the difference between the theoretical and measured SAXS profile. A similar approach can be envisioned for FRET data which can be directly interpreted as a probability distribution on the distance between some donor and acceptor groups, which can be turned into an energy function in the same way as our coarse-grain measures. Other low-resolution methods such as hydroxyl radical footprinting offer information about how accessible a particular nucleotide is to solvent (Tullius and Greenbaum 2005), while multiplexed hydroxyl radical cleavage analysis (MOHCA) yields potential interactions between nucleotides within 25 Å of each other (Das et al. 2008). Each of these can be encoded as a potential and sampled from, yielding an ensemble of structures which conform to the constraints imposed by the experimental method. Given the probabilistic nature of the potentials, uncertainty about the constraints (due to difficulty in resolving gel bands, for example) can be encoded in the target distribution imposed by the experimental data.

Beyond the potential applications, this work aims to provide a platform for further exploration into the determinants of global tertiary RNA structure. The inclusion of predicted local structural motifs (Lescoute et al. 2005; Sarver et al. 2008; Petrov et al. 2013; Theis et al. 2013) provides an immediate avenue for the improvement of the prediction quality. Information about the extended secondary structure (Höner zu Siederdissen et al. 2011) of a sequence could provide a more fine-grain partitioning of the statistics used in generating the proposal distribution. The framework makes it straightforward to add additional energy terms for long-range interactions and thus provides an orthogonal path for determining what information is necessary for the accurate prediction of global RNA structure.

In summary, coarse-grained 3D RNA structures provide a fast, efficient way toward tertiary structure prediction. They also point toward an information mismatch that we aim to fill with future research. In particular, sequence information is only taken into account during the initial graph construction phase, when the skeleton graph is created from predicted secondary structures. Even using this simplified representation, the lowest energy structure are comparable and often better than some of the more fine-grained prediction methods. In addition, Ernwin provides a more thorough and wider sampling of the conformational space than existing methods. Such an accomplishment without sequence information calls into question the efficacy of the sampling approaches of other more fine-grained methods and provides a simplified model for exploring new methods of building and sampling de novo structures.

## SUPPLEMENTAL MATERIAL

Supplemental material is available for this article.

## Supplementary Material

Supplemental Material
