# Supplementary material for: Predicting RNA 3D structure using a coarse-grain helix-centered model
Source: RNA. 2015 Jun;21(6):1110–21. doi: 10.1261/rna.047522.114 (PMC4436664; doi:10.1261/rna.047522.114)
Supplement: Supplemental Material [file supp_21_6_1110__index.html]

Predicting RNA 3D structure using a coarse-grain helix-centered model — Predicting RNA 3D structure using a coarse-grain helix-centered model — Supplemental Material 

# Predicting RNA 3D structure using a coarse-grain helix-centered model

## Supplemental Material

**Files in this Data Supplement:**

- Supp Material.pdf
