# Supplementary material for: Predicting RNA 3D structure using a coarse-grain helix-centered model
Source: RNA. 2015 Jun;21(6):1110–21. doi: 10.1261/rna.047522.114 (PMC4436664; doi:10.1261/rna.047522.114)
Supplement: Supplemental Material [file supp_047522.114_SuppMaterial.pdf]

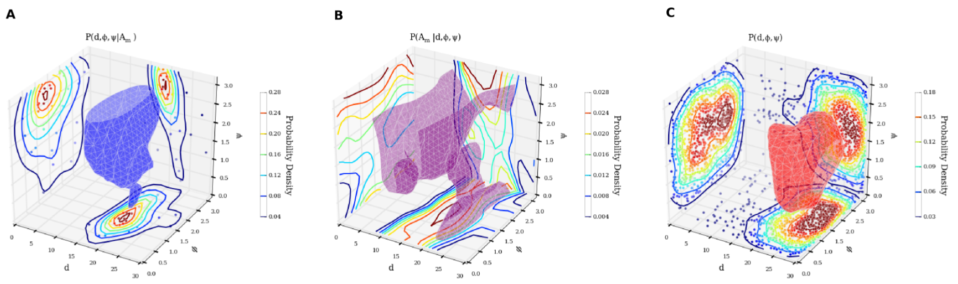

Figure A.1: Cross sections and iso-surface of the A-Minor energy as calculated for interior loop to stem A-minor interactions. (A) The probability density of seeing interaction parameters given an A-Minor interaction. (B) The probability density of an interaction as calculated using Bayes' law. (C) The probability density of seeing a particular set of parameters among all adenine-containing hairpin loops within 30 Å of each other. The iso-surface in each plot corresponds to the mean probability density of all the points on the 3D grid describing the parameter space.

A. Supplementary Material

A.1. Energies

A.1.1. Clash Energy

Having established that we can re-position the atoms of a helix reasonably well given its coarse grain parameters (see Sec. 2.2), we can use this technique to detect clashes within stem regions. This is done by 'virtual' base pairs, or interpolations of where a base pair and its constituent nucleotides and atoms would lie given its position within the stem and the stem's coarse-grain parameters.

Thus, if a stem has a length of n base pairs, a starting position vector s, an ending position vector e and two twist vectors t_1 and t_2, the position of the i'th virtual base pair along the axis of the helix will be $s + \frac{i-1}{n-1}(e - s)$. The direction of the angular position can be calculated in a similar fashion by taking the corresponding proportion of the angle that one would need to rotate the first twist around the stem axis to align it with the second twist. This total angle, naturally, needs to be adjusted for helices in which the base pairs twist fully around the stem axis one or more times (usually around the 11th basepair).

Using the stem axis vector $a = (e - s)$, the starting position of the virtual base pair v_s, and the virtual twist vector v_t, we can define a coordinate system for each base pair. Within this coordinate system, the position of all the atoms of the two nucleotides in the base pair can be represented in a manner that only depends on the strand which contains the nucleotide and the identity of the atom. These positions were calculated for all stem-contained atoms in the ribosome structure `1jj2` and averaged to create an average base-pair representation.

The clash energy function determines if the positions of the atoms calculated from the virtual base-pairs of each helix intersect (i.e. are positioned within 1.8 Åof each other). The number of intersections is multiplied by a large energetic penalty to yield a certain rejection of the sampled structure.

A.1.2. A-Minor Energy

A.2. Best Sampled Structures

A.3. Enumeration

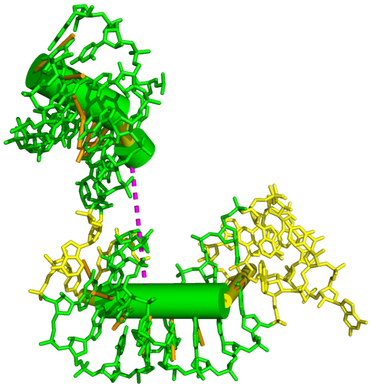

Figure A.2: An example of an A-Minor interaction where the line between the closest points on the two coarse-grain elements (dashed purple line) does not exactly correspond to the location of the interaction, which is on the far left of end of the bottom helix.

Table A.1: The RMSD values and best models produced by each prediction method. Missing structures indicate an error in the prediction pipeline or program.

PDB ID	Length	Ernwin	RNAComposer	FARNA	NAST
3FO4	63	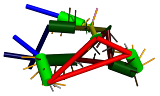<br>6.7	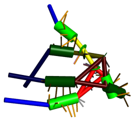<br>7.7	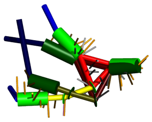<br>4.6	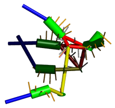<br>10.2
3R4F	66	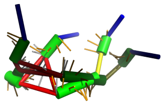<br>12.6	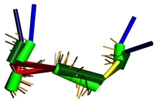<br>3.1	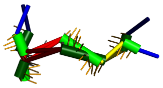<br>4.5	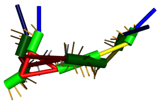<br>6.1
4PQV	68	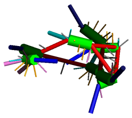<br>11.3	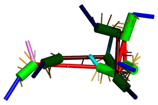<br>10.4	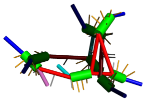<br>9.0	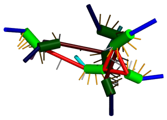<br>10.5
1YFG	69	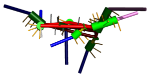<br>8.2	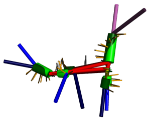<br>2.1	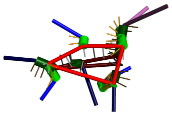<br>8.9	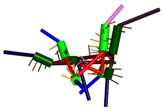<br>10.3

Continued on next page

Table A.1 – continued from previous page

PDB ID	Length	Ernwin	RNAComposer	FARNA	NAST
1FIR	69	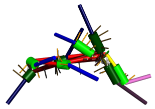<br>9.1	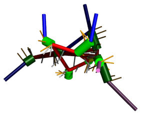<br>14.0	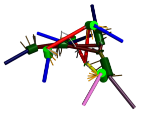<br>10.3	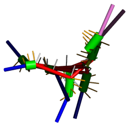<br>10.5
1KXK	70	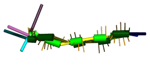<br>5.3	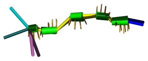<br>1.1	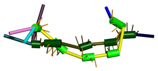<br>8.7	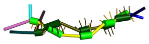<br>5.2
1Y26	71	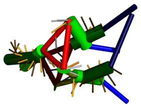<br>5.6	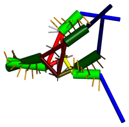<br>6.1	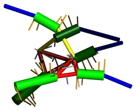<br>10.0	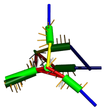<br>9.2
2TRA	73	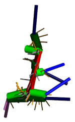<br>7.8	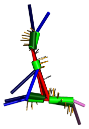<br>1.6	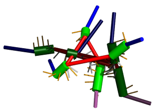<br>12.2	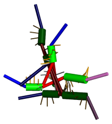<br>14.6
3CW5	75	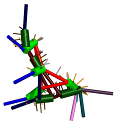<br>7.3	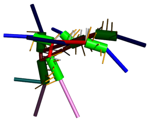<br>8.1		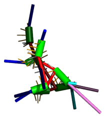<br>9.8
2HOJ	78	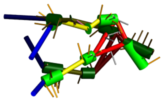<br>7.1	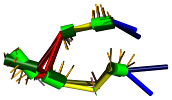<br>1.9	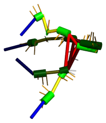<br>11.1	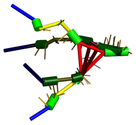<br>7.8
4P5J	83	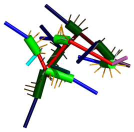<br>14.1	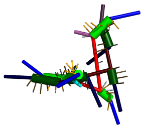<br>9.0	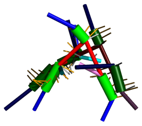<br>10.9	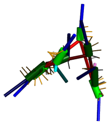<br>11.5
3T4B	83	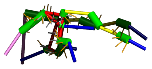<br>7.4	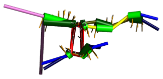<br>2.3	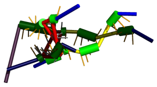<br>11.2	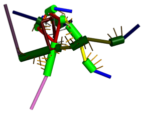<br>13.4

Continued on next page

Table A.1 – continued from previous page

PDB ID	Length	Ernwin	RNAComposer	FARNA	NAST
4FRG	84	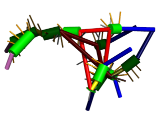<br>14.5	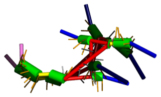<br>5.9	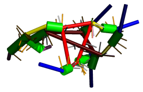<br>12.3	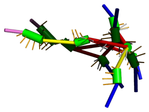<br>12.8
4LVZ	89	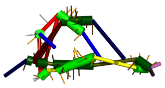<br>9.2	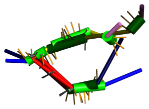<br>7.7	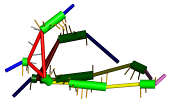<br>10.5	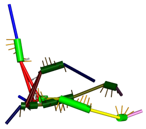<br>18.8
3GX5	94	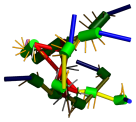<br>16.8	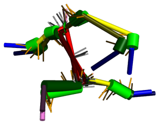<br>2.9	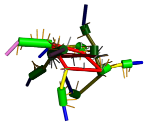<br>22.0	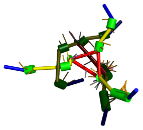<br>11.6
4L81	96	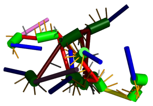<br>10.1	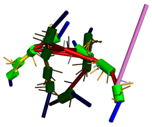<br>16.5	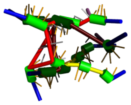<br>13.6	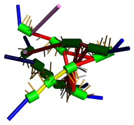<br>10.6
2QBZ	153	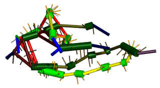<br>19.5	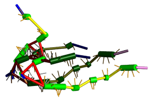<br>28.9	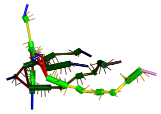<br>30.9	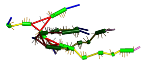<br>35.1
1U9S	155	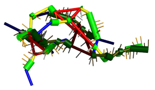<br>15.0	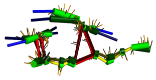<br>3.0	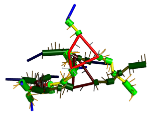<br>18.8	
1GID	158	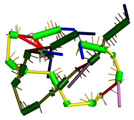<br>25.2	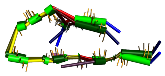<br>3.8	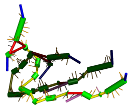<br>24.3	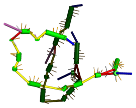<br>28.8
3D0U	161	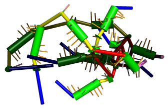<br>23.3	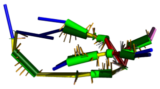<br>2.1	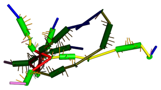<br>20.3	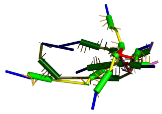<br>15.6

Continued on next page

Table A.1 – continued from previous page

PDB ID	Length	Ernwin	RNAComposer	FARNA	NAST
4GXY	161	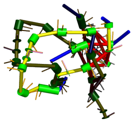 16.0	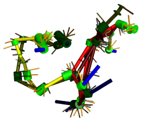 9.5		
3DIR	172	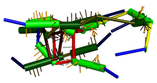 21.6	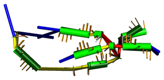 1.6	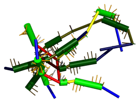 24.9	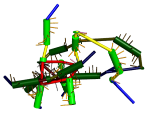 25.1
4P8Z	188	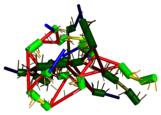 21.6	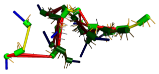 27.2	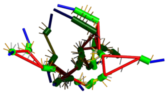 23.5	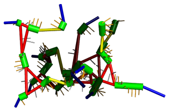 24.3
4P9R	189	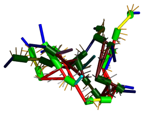 14.7	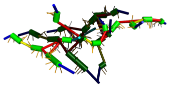 29.4	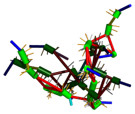 26.0	
4GMA	192	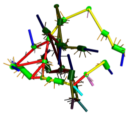 24.8	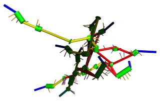 32.7	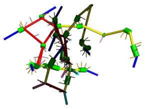 38.2	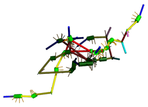 38.5
3DHS	215	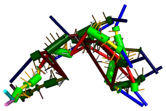 19.9	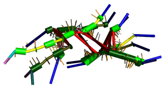 9.0	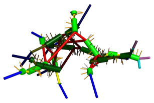 25.2	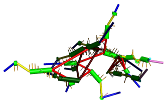 29.6
1X8W	242	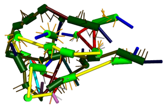 27.1	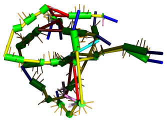 24.6	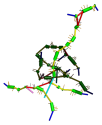 45.7	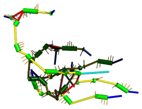 37.7
2A64	298	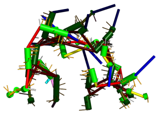 25.4	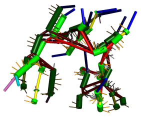 20.9		

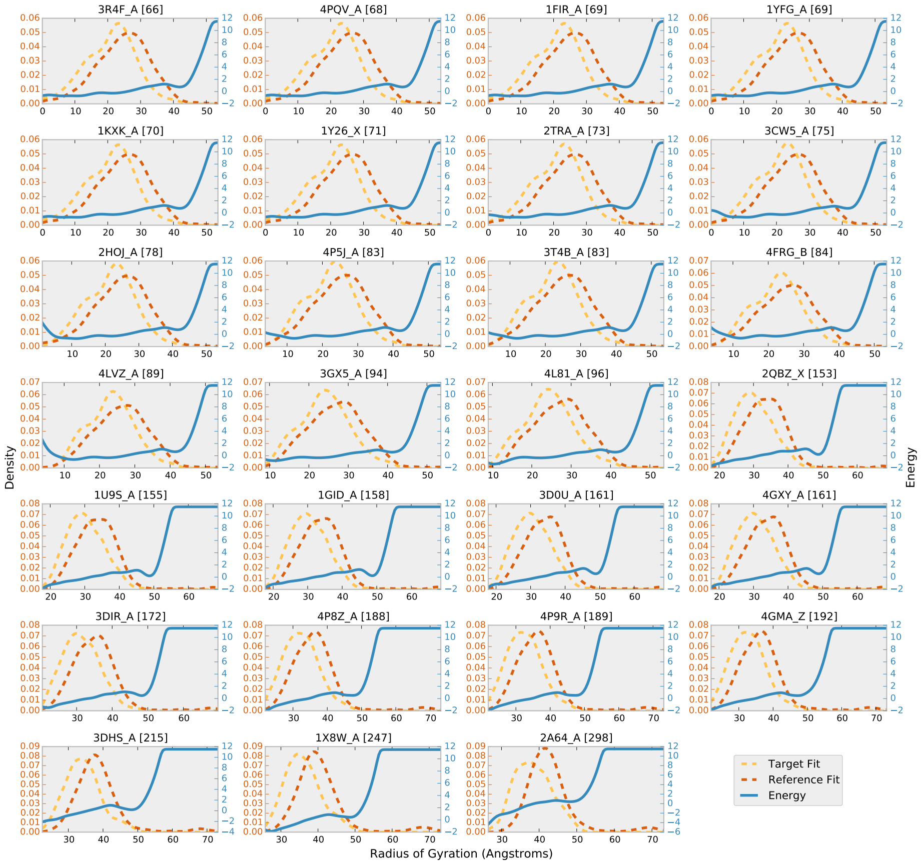

Figure A.3: The target and reference distributions for the radius of gyration. The target and reference measures are obtained from subgraphs of a native ribosome and an artificially constructed structure without clashes or broken junctions. Clearly evident is the tendency for native structures to have a smaller radius of gyration than larger structures. The energy values are thus lower for more compact structures and higher for more spread out structures. The effect becomes more pronounced for larger structures due to their inherent ability to spread out more.

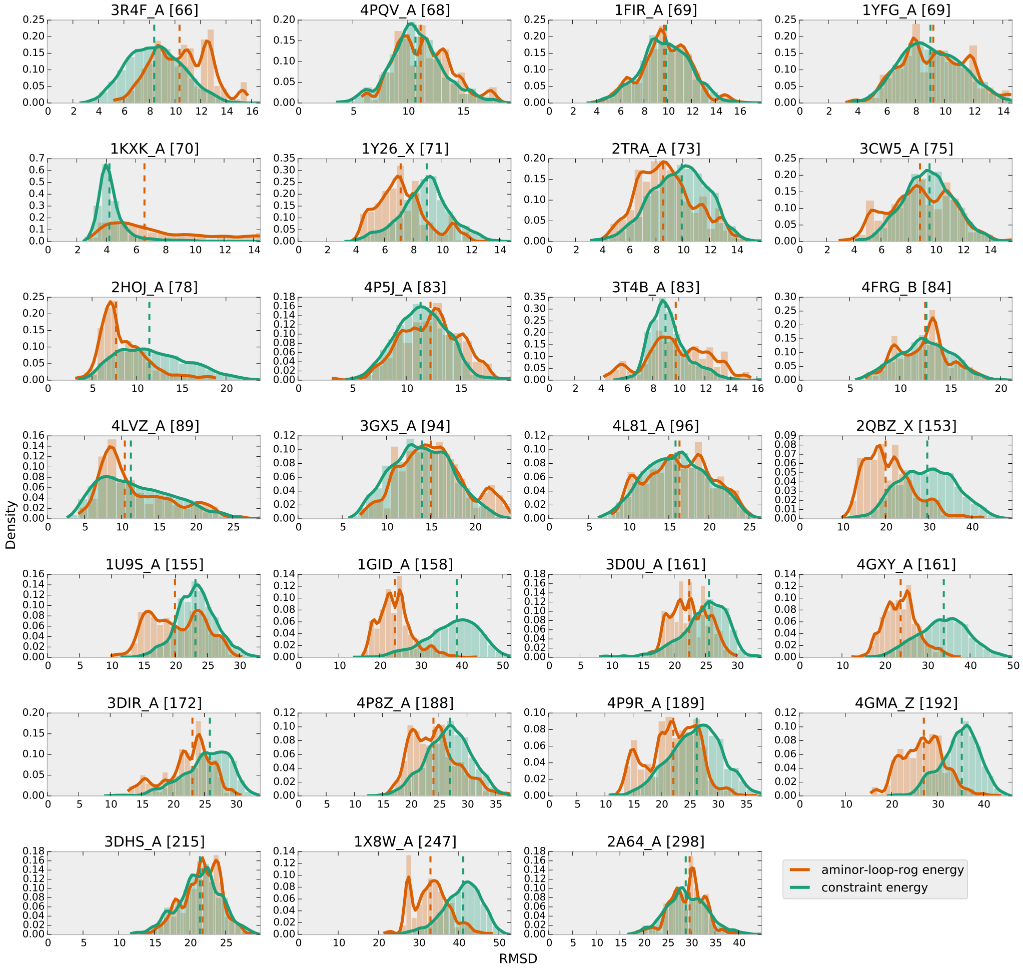

Figure A.4: The distribution of the RMSD between the native and the sampled structures using a naive (constant-energy) energy function and the combination of the three non-constraint coarse grain energies (aminor-perloop-rog).

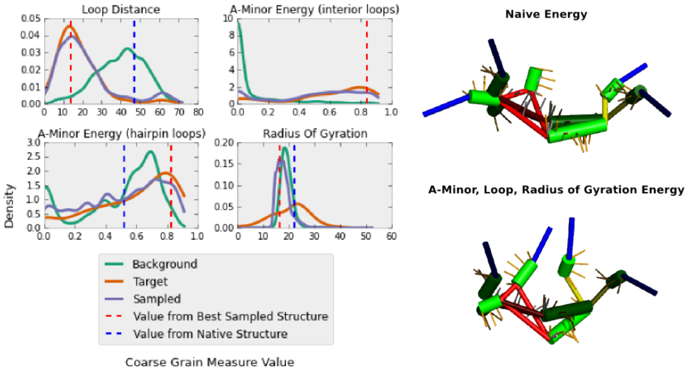

Figure A.5: The best model for the structure 3R4F (bright colors) aligned to the native structure (dark colors) from a simulation using the naive energy function (above right) and the energy described in Section 2.4 (below right). On the left are the graphs indicating the coarse grain value distributions for each of the measures. The value of the best sampled structure is shown as an orange dashed line, whereas the value from the native is shown as a dashed blue line. The two lines show a perfect overlap for the interior loop A-Minor Energy in the upper right hand corner and thus only the red line is visible.

A.4. Example of a Prediction Failure

As seen in figure A.4, sampling using the presented energy function does not always lead to an improvement over a naive energy. A good example of this is the structure 3R4F, show in Supplementary Figure A.5. The poor prediction performance is most likely attributed to the incorrect close positioning of the two hairpin loops in the predicted structure. The other coarse-grain values differ by smaller magnitudes and likely contribute less to the wrong configuration. Examining the energy of all of the sampled structures show that this structure has a very unfavorable energy-rmsd profile (see Supplementary Table A.2). This example illustrates one of the pitfalls of using a knowledge-based energy function wherein a strong pattern in the training data is not necessarily reflected in individual structures. We plan on addressing this issue by adding additional terms to our energy function such that a global maximum probability (minimum energy) occurs near the native structure. This, however, is an ongoing pursuit and is beyond the scope of this paper.

A.5. Helix Fitting

Four different methods for fitting a coarse grain helix (consisting of an axis segment and two twist vectors) to an all-atom helix were tested for their ability to accurately represent the positions of the atoms on the helix.

The **ad-hoc** method uses the directions of the base normals as well as the vector between the estimated centers of the outermost two base pairs of the helix as a way of calculating the axis vector.

The **fit** method assumes that the projection of the positions of backbone atoms onto the plane normal to the axis vector should form a circle. The axis is calculated by optimizing its vector so as to minimize the root-mean square deviation (RMSD) between the projected heavy atom positions and a circle fit onto their positions.

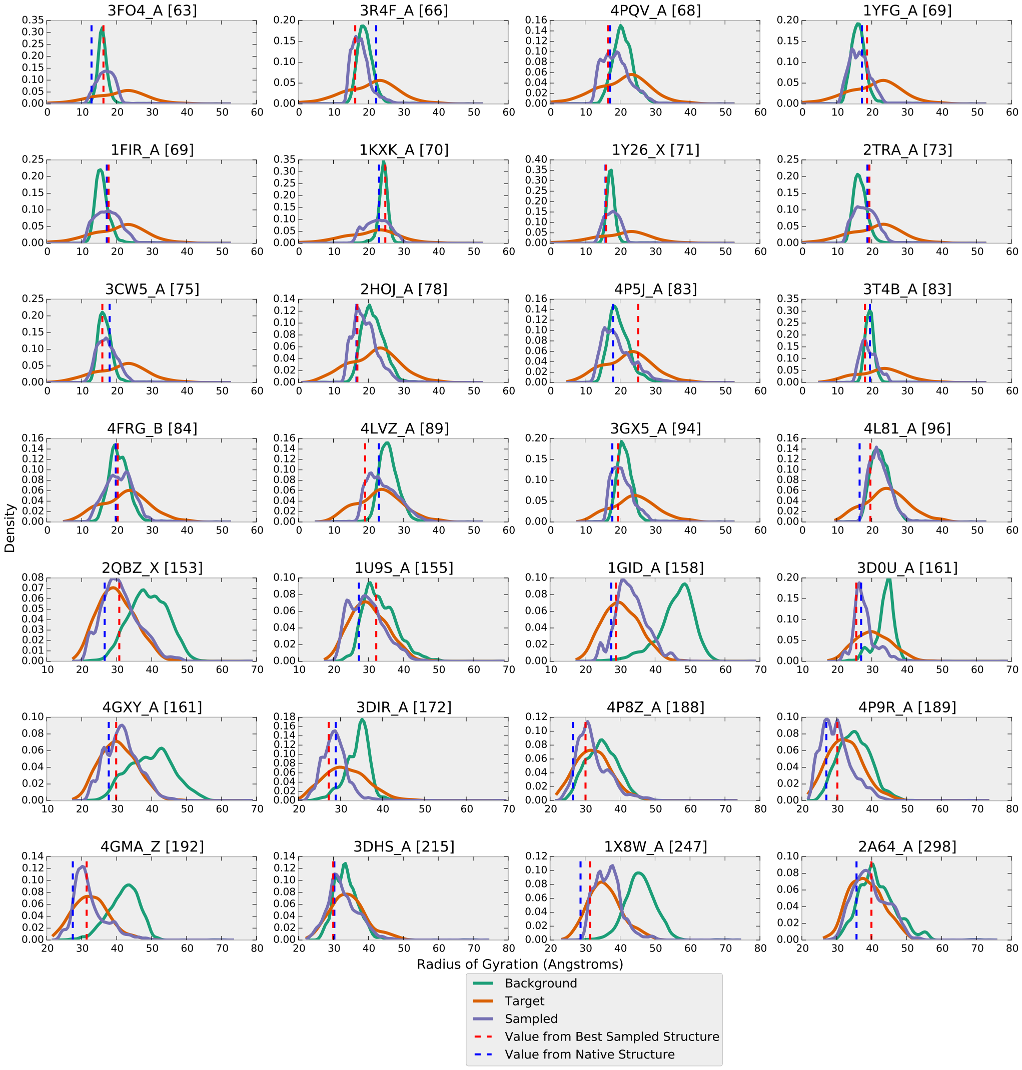

Figure A.6: The distribution of the radius of gyration values for structures derived from the native ribosome (target, orange), the naive sampling of the modeled structure (background, green) and the energy-directed sampling of the modeled structure (sampled, purple).

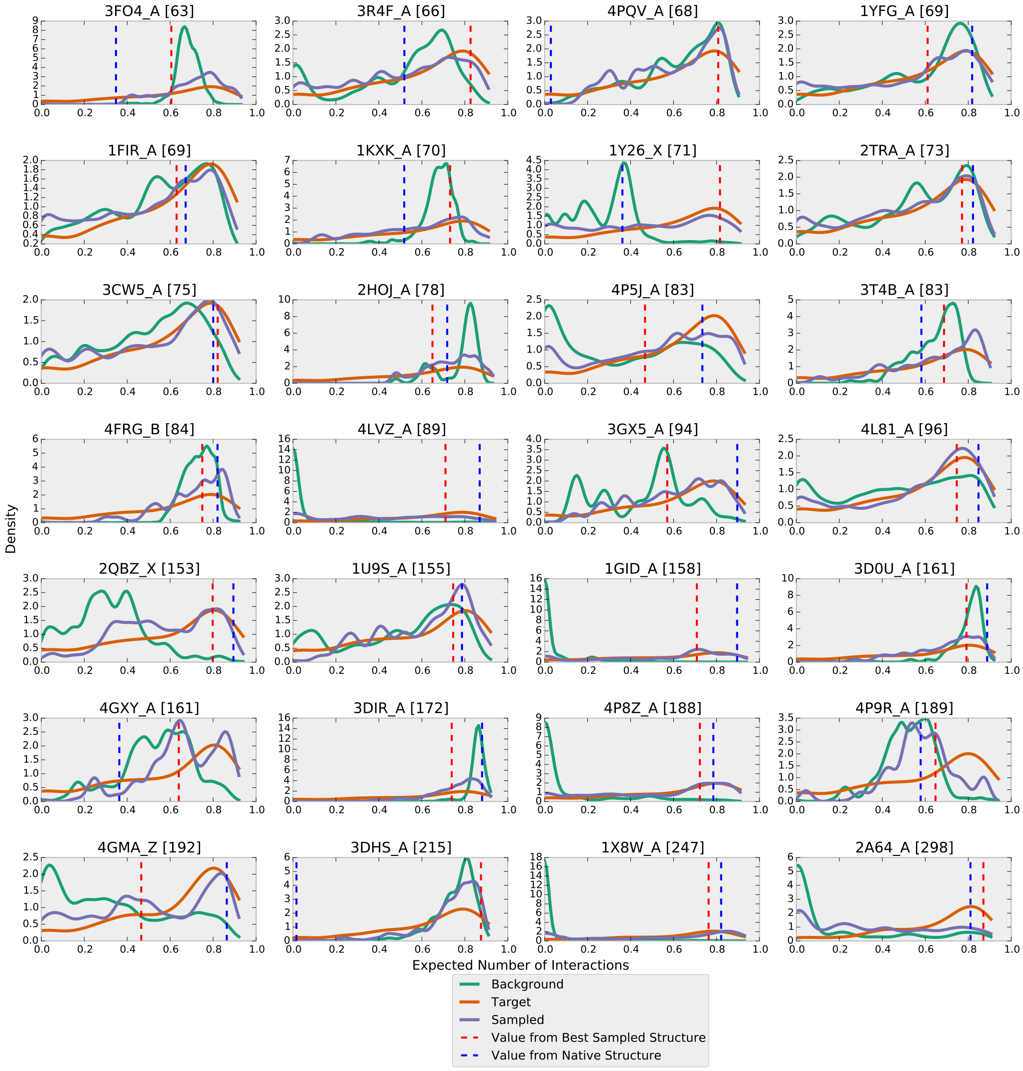

Figure A.7: The distribution of A-Minor interaction probabilities for hairpin loops for structures derived from the native ribosome (target, orange), the naive sampling of the modeled structure (background, green) and the energy- directed sampling of the modeled structure (sampled, purple).

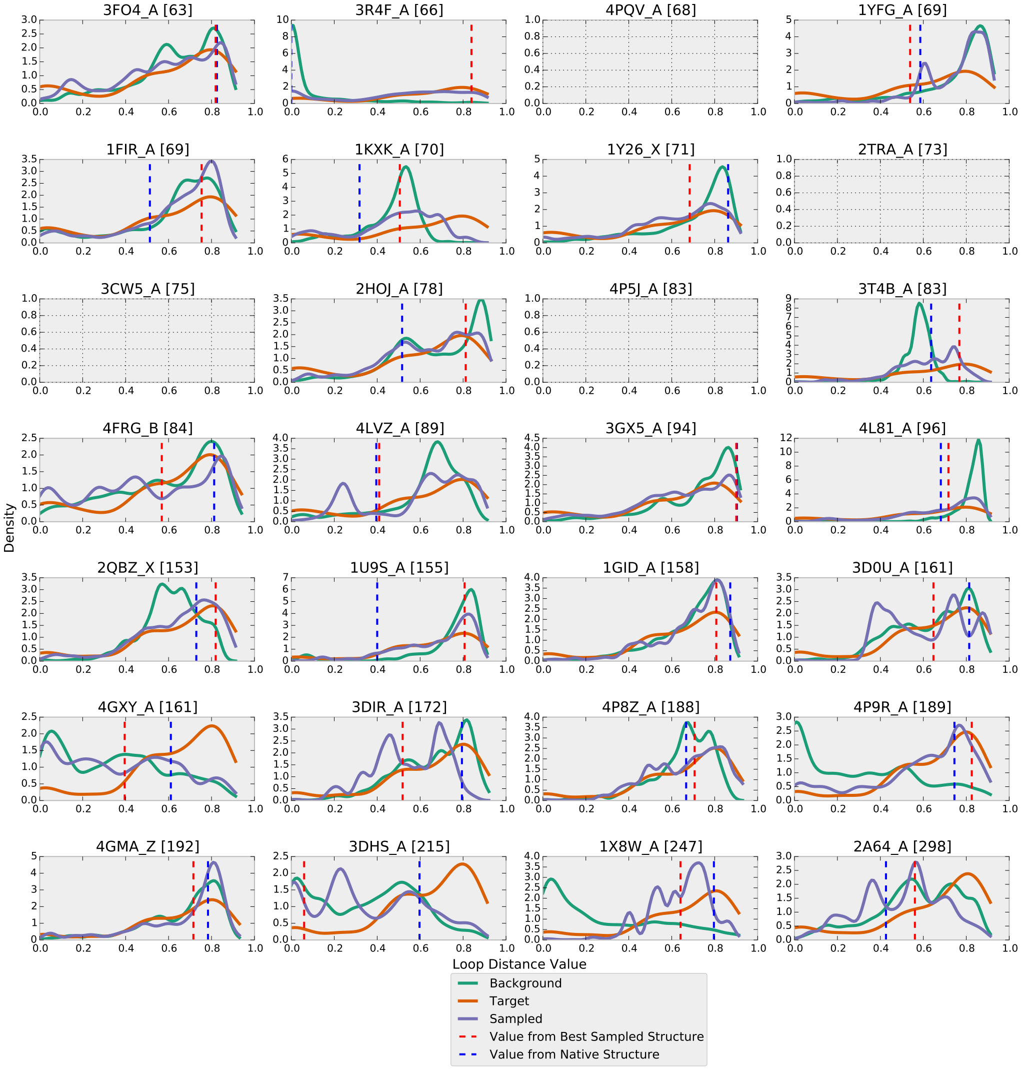

Figure A.8: The distribution of A-Minor interaction probabilities for interior loops for structures derived from the native ribosome (target, orange), the naive sampling of the modeled structure (background, green) and the energy- directed sampling of the modeled structure (sampled, purple).

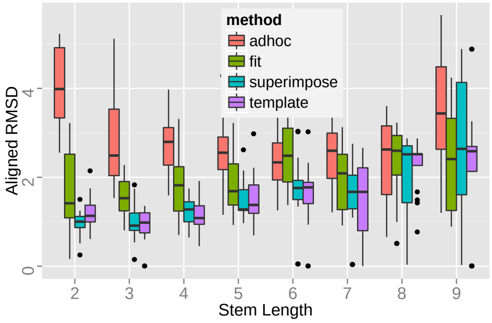

Figure A.9: A comparison of the ability of the helix-fitting methods to re-orient a different helix onto the original helix, given only the fitted helix parameters. The difference between the different methods is most pronounced in shorter stems which contain the least amount of data points available for helix-fitting. For the best method, the average aligned root mean square deviation increases for larger stems due to the increasing number of nucleotides present.

The **superimpose** method fits an axis as per the **fit** method onto an ideal helix (generated by using the fiber program of the 3DNA package [25]). The ideal helix is then superimposed onto the target helix using Kabsch's algorithm [17] and the resulting transformation is applied to the fitted axis to yield an axis for the target helix.

The **template** method differs from the **superimpose** method insofar as it uses an extremely long (30 base pair) helix to fit the axis parameters and then superimposes the first n base pairs of the long ideal helix onto the target helix to generate the transformation for the axis.

A.5.1. Results

To measure the quality of each helix fitting method described in the previous section, we took pairs of helices containing the same number of base-pairs and calculated the parameters for both helices using each of the four described methods. For both helices, we calculated a twist parameter equal to a halfway rotation of one twist onto the other around the helix axis. The direction of this twist parameter as well as the line segment defining the axis of the helix provide enough parameters to direct the superposition of one helix onto another. That is, we can define a translation and a rotation that will superimpose one coarse grain representation onto another. By applying this transformation to each backbone atom of the second helix, we attempt to move and rotate it to its equivalent location on the first helix. We would expect a consistent helix fitting method to yield the lowest RMSD deviation between the atoms of the first helix and the atoms of the second (fitted) helix when superimposed on the first using the axes and twist parameters defining both helices.

The results (illustrated in Fig. A.9) confirm the expected dominance of the superposition-based methods (**superimpose** and **template**). The ad-hoc method's performance on short helices was notably worse than any of the others due to the fact that the conformations of terminal base pairs tend to have a variable geometry which is not tempered by the length of the helix. In longer helices, the vector between the computed centers of the terminal base pairs is longer and thus has less room for variation, leading to better performance of the ad-hoc method. The **fit** method, which fits a helix axis by optimizing

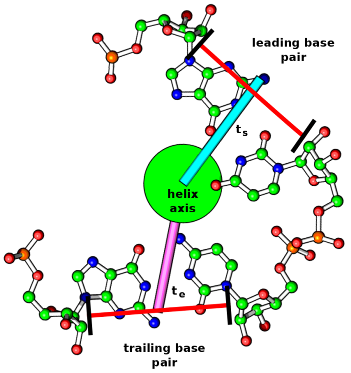

Figure A.10: An illustration of a fitted cylinder along with the 'twist' vector indicating the direction of the first and last base pair of the stem. The view is top-down looking down the axis of the stem. The red segments indicate the vectors between the C1' atoms of the first and last base pairs. The point bisecting these segments provides the direction of the 'twist' vectors.

the mean error of a circle fit to the projection of the backbone atoms onto the plane perpendicular to the axis, also lags behind the superposition based methods on shorter helices. The paucity of data points (backbone atoms) leads to semi-circular and noisy projections which in-turn lead to potentially inaccurate circle fits. The two superposition-based methods show almost identical performance due to the regular nature of the ideal helices to which the axis and circle are fit. The quality of all methods degrades slightly for larger helices due to the increased tendency of helices to bend slightly and deviate from the ideal geometry as they increase in length.

A.5.2. Ad-hoc

Let the residues on one strand of the helix be numbered s_{a_1},, s_{a_2}, ..., s_{a_n}, and on the other strand s_{b_1},, s_{b_2} , ... , s_{b_n}, where $s_{a_{n-1}} < s_{a_n}$ and $s_{b_{n-1}} < s_{b_n}$ then the two residues that cap one end of the helix are s_{a_1} and s_{b_n} while the residues that cap the other end of the helix are s_{a_n} and s_{b_1}. Let $c_\alpha(s)$ define the location of the C_α atom in the residue numbered s. The helix vector which is used as an initial estimate for the optimization function described above is calculated as follows:

$$
\begin{aligned}
V_{s1} &= c_\alpha(s_{a_1}) - c_\alpha(s_{b_n}) \\
V_{s2} &= c_\alpha(s_{a_1} + 1) - c_\alpha(s_{b_n} - 1) \\
V_{e1} &= c_\alpha(s_{a_n}) - c_\alpha(s_{b_1}) \\
V_{e2} &= c_\alpha(s_{a_n} - 1) - c_\alpha(s_{b_1} + 1)
\end{aligned}
$$

The vectors V_{s1}, V_{s2}, V_{e1}, and V_{e2} are between the C_α atoms of two base-paired nucleotides. They should be roughly orthogonal to the axis of the helix.

$$S_n = V_{s_1} \times V_{s_2}$$
$$E_n = V_{e_1} \times V_{e_2}$$
$$S_y = S_n \times V_{s_1}$$
$$E_y = E_n \times V_{e_1}$$

The vectors S_n and E_n should be two estimates of the helix axis as calculated from the top and bottom two nucleotides. S_y and E_y should be orthogonal to the estimated helix-axis as well as the vector between the first and last C_α atom of the helix, respectively.

$$S_c = (\frac{V_{s1}}{|V_{s1}|} + \frac{S_y}{|S_y|})/2$$
$$E_c = (\frac{V_{e1}}{|V_{e1}|} + \frac{E_y}{|E_y|})/2$$
$$S = c_\alpha(s_{a_1}) + \frac{8.4 \cdot S_c}{|S_c|}$$
$$E = c_\alpha(s_{b_1}) + \frac{8.4 \cdot E_c}{|E_c|}$$
$$V_{est} = E - S$$

Taking the average of the normalized C_α vectors and the previous orthogonal vector should yield a vector roughly towards the center of the helix. Finally, adding a multiple of this vector to the C_α atoms of the start and end residues, yields rough estimates for the start and end of the helix. This is the crudest method of estimating the cylinder axis vector and performs consistently worse than the other methods (figure A.9).

A.5.3. Fit Method

An improvement over this method comes from the realization that a helix is a path along a cylindrical manifold. We proceed by fitting a cylinder to the backbone atoms of an RNA helix. Given a cylinder with an axis vector C_v, then a coordinate system can be created with the z-axis lying along C_v. Assuming the axis vector defines a cylinder with radius r which minimizes the root- mean-square distance of the backbone atoms from the surface of the cylinder, then transforming the locations of the backbone atoms into the coordinate system defined by the cylinder vector should yield a circle on the plane defined by the x and y axes which has a minimum root-mean-square deviation from the circle created by intersecting our ideal cylinder with the x-y plane of the our transformed coordinate system. These assumptions yield a straightforward method of fitting a cylinder to an RNA helix by fitting an axis such as to minimize the root-mean-square deviation of the best circle that can be fit onto the transformed backbone atoms of the RNA helix. The use of this method significantly improves the ability to align a second helix onto the first over the naive 'estimate' method (Fig. A.9).

Fitting the circle is done via the method described by [4], while fitting the axis is done using Python's leastsq optimization function. An initial estimate for the axis vector is calculated using the ad-hoc method.

A.5.4. Superimpose and Template Methods

RNA helices, especially of the shorter variety, often vary slightly in their structure such that their atoms do not form an ideal circle when viewed along the helix axis. To compensate for this, we first created an idealized stem using the fiber program [25], superimposed it onto the stem being parameterized and then calculated the cylinder axis for the ideal stem. The resulting method (called **superimpose**) showed a marked improvement over all of the previous attempts at approximating a helix.

Longer helices, as one may expect, should be easier to parameterize due to the larger number of data points available. The final method we tried was to create an idealized 30 base-pair helix and to approximate its axis using the 'fit' method described above. For every subsequent helix of length n ($n < 30$) for which we wanted to calculate an axis, we simply calculated the best rotation and translation to align its atoms onto those of the first n base pairs of long reference helix. The resulting transformation was applied to the reference axis which was further cropped to match the length of the query helix. The results indicate that while this method (called **template**) was more consistent, the parameters it created were on average no better than those created by the 'superimpose' method (Fig. A.9). Nevertheless, it is the method that is used throughout this paper for fitting coarse grain helices onto all-atom models.

A.5.5. Twist Parameters

In addition to the cylinder defining the helix, we need an approximation for how the base pairs are positioned along this cylinder. This is accomplished simply by storing vectors (henceforth called twist vectors) which indicate the direction from each end of the cylinder to the middle of the terminal base pairs. If the axis of the stem cylinder is defined by a start point, C_s, and an end point, C_e, then the twist vectors are calculated by the taking the vector rejection of a, (the average of the vectors from the end of the cylinder to the terminal nucleotides' C_α atoms), from the cylinder's direction vector.

$$\begin{aligned} a &= c_\alpha(s_{a_1}) - C_s + (c_\alpha(s_{b_n}) - C_s) \\ t &= a - \left(\frac{a \cdot V}{V \cdot V}\right) V \end{aligned}$$

A.6. Sampling Quality and Energy Factor

A.6.1. Energy Function Evolution

As the sampling proceeds, the distribution of the sampled values for each coarse grain measure change and the reference distribution for the energy function must also change. This is starkly illustrated in the case of the adenine riboswitch (PDB: `1Y26`). The initial energy function, as calculated from substructures of the native and artificially constructed ribosome (PDB: `1jj2`), favors more compact structures due to the relative paucity of such structures in the initial reference distribution (see Fig. A.3, upper left hand plot labeled `1Y26`). If, however, we look at conformations sampled for this particular secondary structure (as opposed to the collection of ribosome substructures of roughly the same size), it becomes apparent that the background distribution actually favors more compact structures than the target distribution (see green and orange, respectively in Fig. A.6, upper left corner labeled `1Y26`). Thus as the sampling proceeds, the energy values for more compact structures stay stable whereas the energy values for more spread out structures decrease (see Fig. A.11).

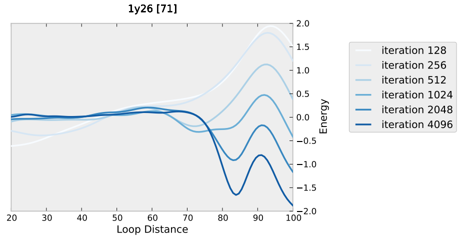

Figure A.11: The change in the values of the energy function as the sampling progresses. With few samples, more compact structures are favored. As more of those accumulate, more spread out structures assume the lower energy values facilitating the sampling of structures matching the target distribution.

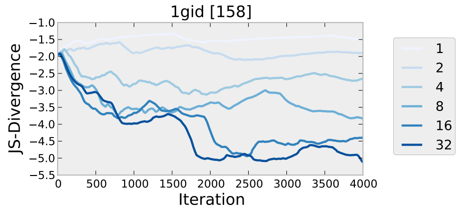

Figure A.12: The Jensen-Shannon divergence between the target and sampled distribution at various points during the simulation. The color of the lines corresponds to the value of the energy weight. Larger energy weights lead to closer matches between the target and sampled distributions and thus lower KL values.

A.6.2. Energy Factor

The energy for each coarse grained value (x) is calculated as the log of the ratio of the target distribution $(p_t(x))$ divided by the reference distribution $(p_r(x))$ and multiplied by a factor c which serves as a parameter for tuning how closely the target distribution should match the sampled values.

$$E = -c \cdot log \frac{p_t(x)}{p_r(x)} \tag{A.1}$$

Since the reference distribution is re-calculated every ten sampling steps, it tends to converge toward the target distribution. Under-sampled and over-sampled regions of the conformation space will contain structures whose energy values have large absolute values. For over-sampled regions, these values will be large and lead to a quick traversal into a different region of the conformational space whereas in under-sampled regions the energy values will be low, yielding more structures in this region. The factor c in the energy formulation above determines how strictly the sampled distribution should follow the target distribution.

The measure of the Jensen-Shannon (JS) divergence between the target and sampled distributions provides a natural measure of when to terminate the sampling. While other methods sample a fixed number of steps in the hope that a sufficiently low energy structure is found, we can simply identify when the JS divergence stabilizes and return the most frequently sampled conformations at that point. Fig. A.13, shows how the JS divergence between the sampled and target distributions varies as a function of how many sampling

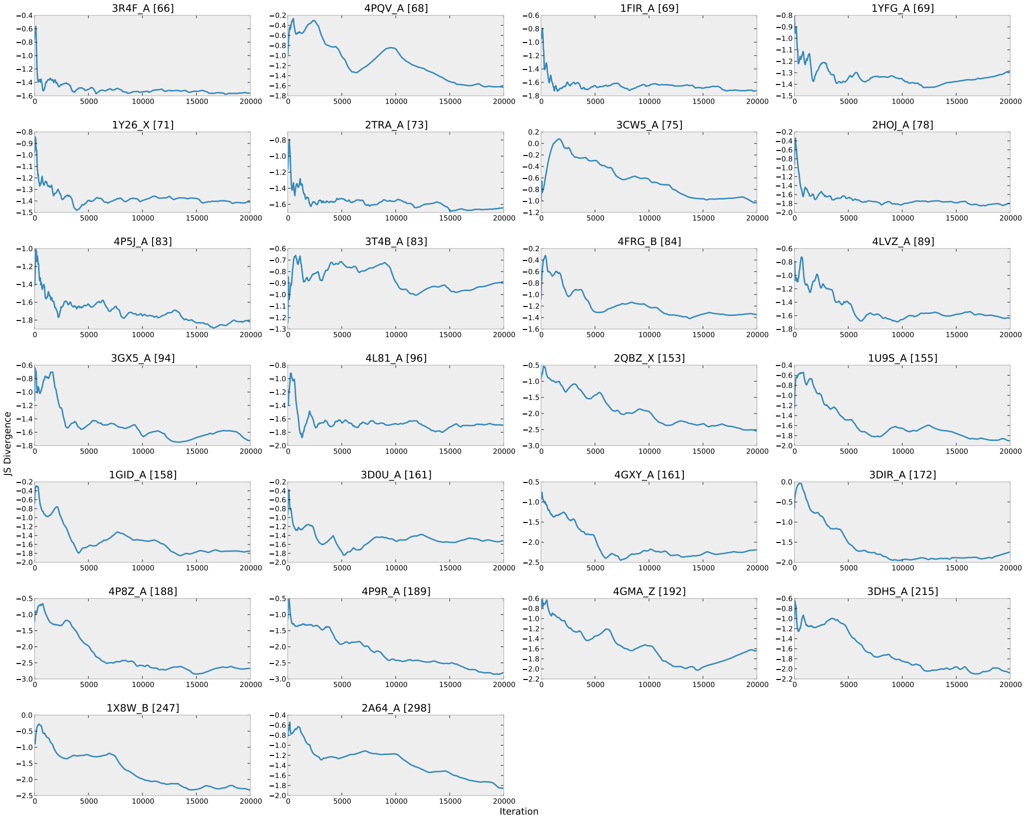

Figure A.13: The Jensen-Shannon divergence between the target and sampled distribution at various points during the simulation for all of the structures tested.

iterations have been performed. We present this merely as an avenue for further research. In actuality, all of our simulations were run to 20000 sampling steps.

A.7. Termination Criterion and Sampling

Table A.2: Energy landscape and profile over the course of an Ernwin simulation. See Main Text Figure 9 for a more detailed description.

3R4F [66]

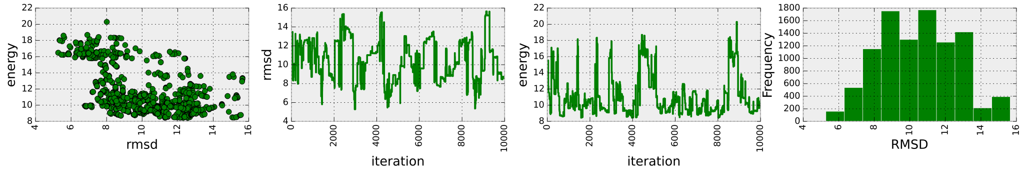

4PQV [68]

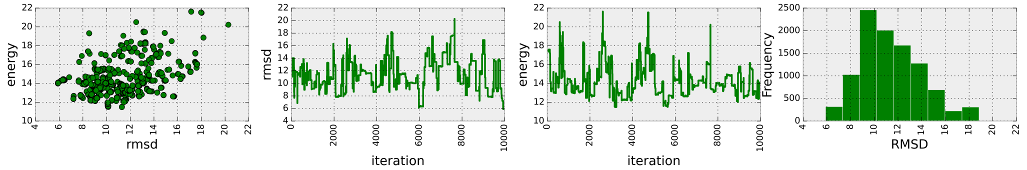

1FIR [69]

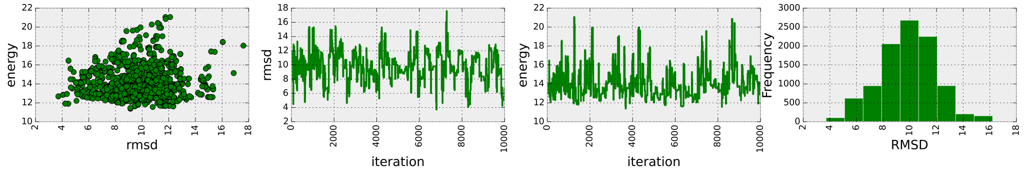

1YFG [69]

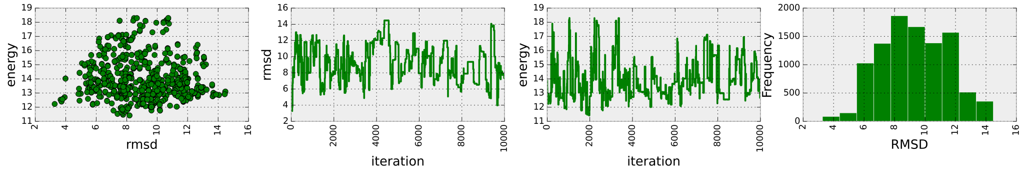

1KXK [70]

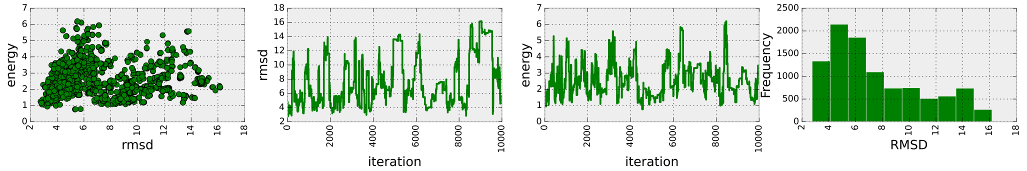

1Y26 [71]

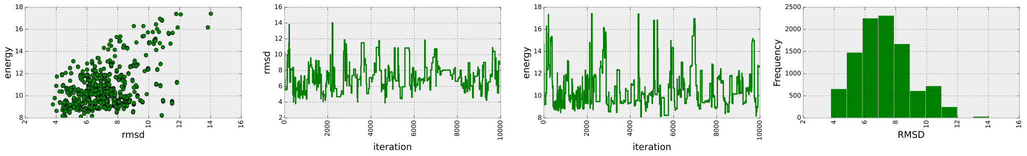

Continued on next page

Table A.2 – continued from previous page

2TRA [73]

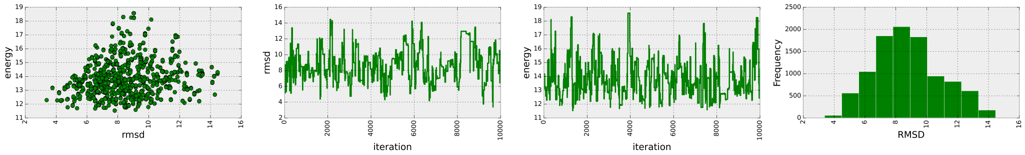

3CW5 [75]

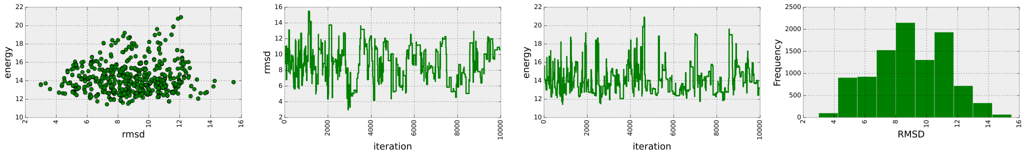

2HOJ [78]

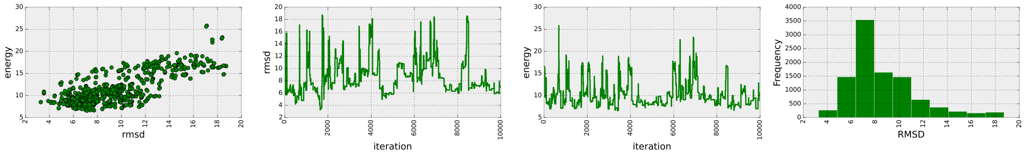

4P5J [83]

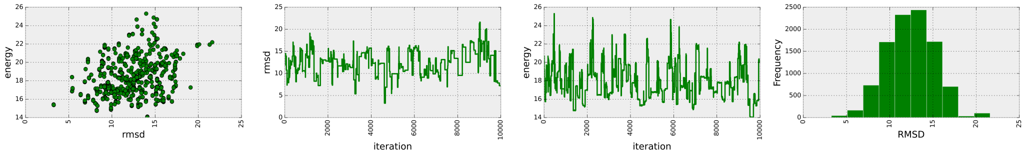

3T4B [83]

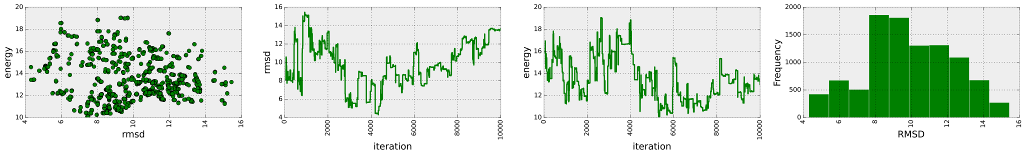

4FRG [84]

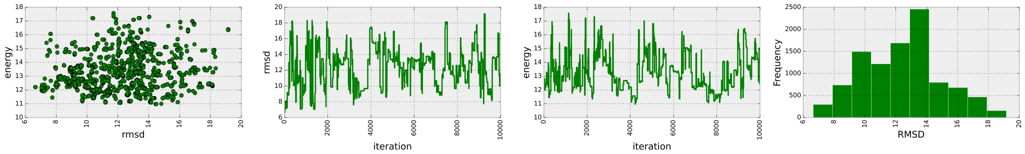

4LVZ [89]

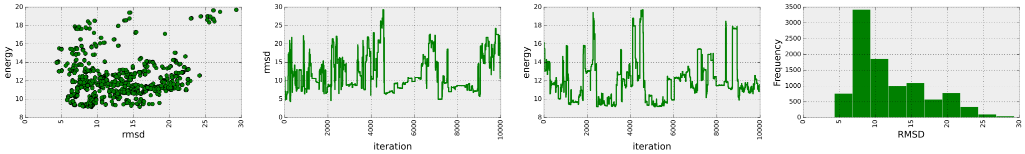

Continued on next page

3GX5 [94]

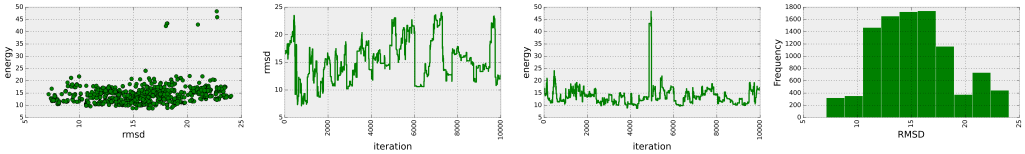

4L81 [96]

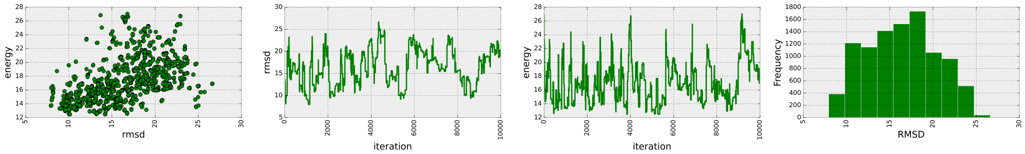

2QBZ [153]

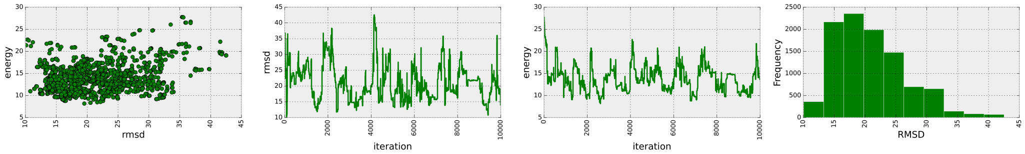

1U9S [155]

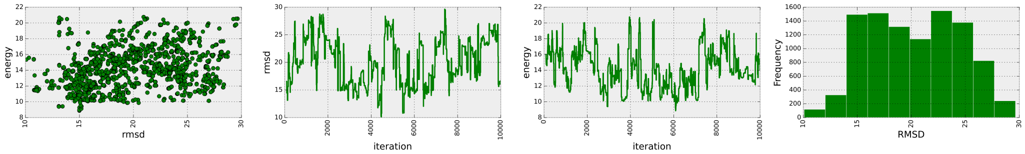

1GID [158]

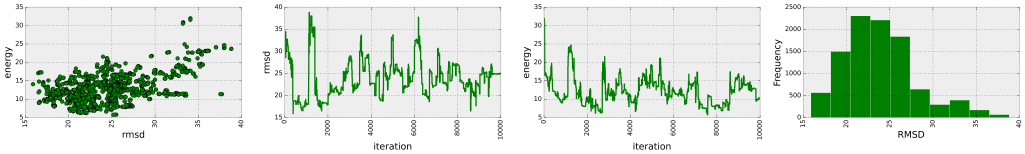

3D0U [161]

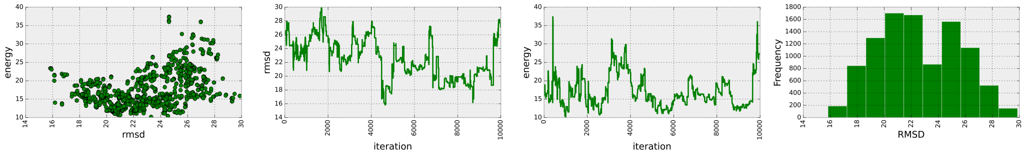

4GXY [161]

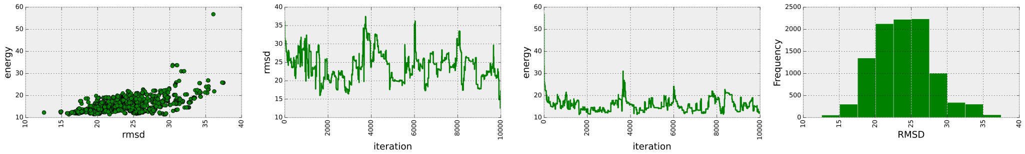

Continued on next page

3DIR [172]

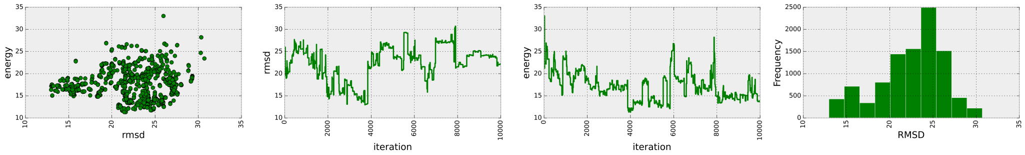

4P8Z [188]

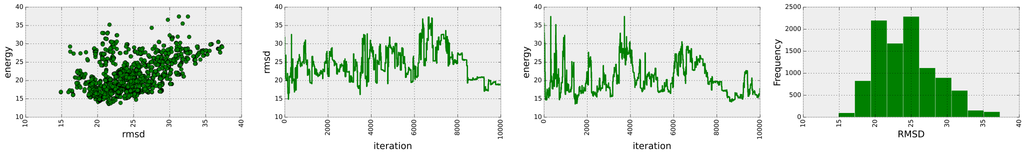

4P9R [189]

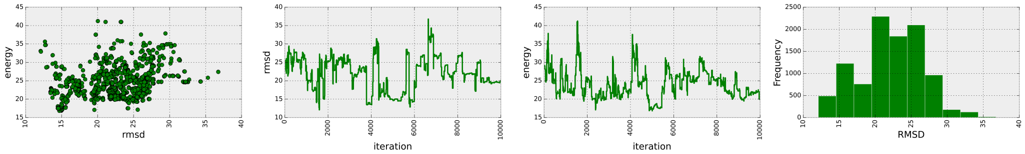

4GMA [192]

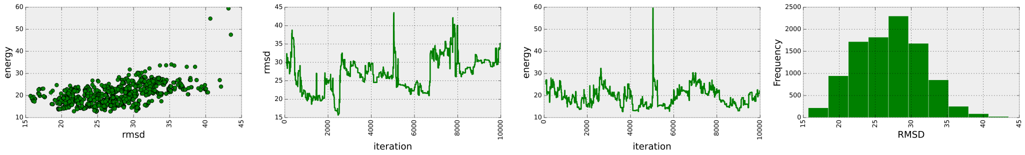

3DHS [215]

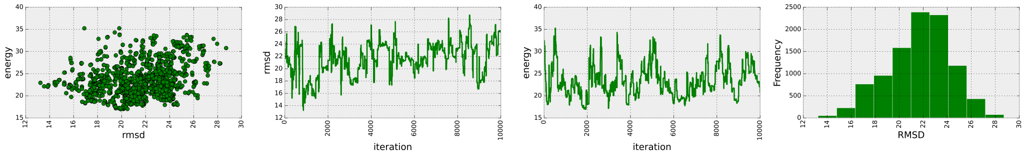

1X8W [247]

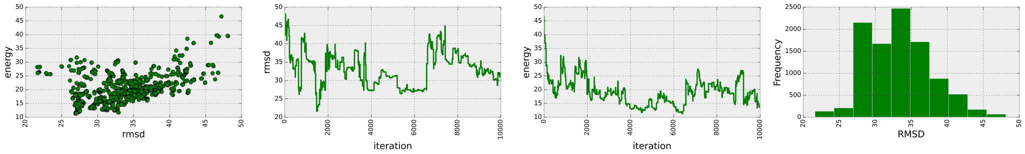

2A64 [298]

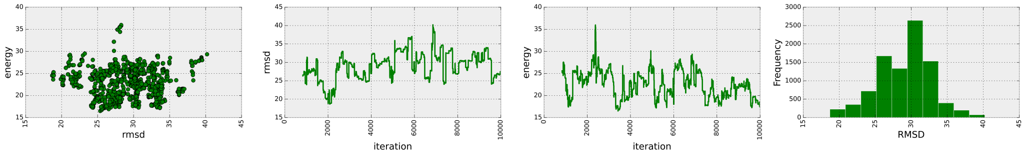

Table A.3: Energy landscape and profile over the course of an Ernwin simulation. See Main Text Figure 9 for a more detailed description.

3R4F [66]

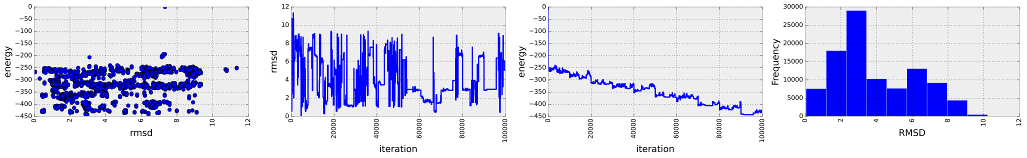

4PQV [68]

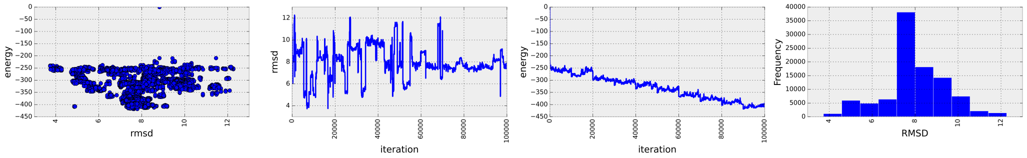

1FIR [69]

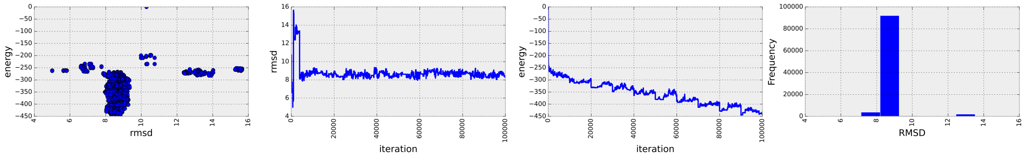

1YFG [69]

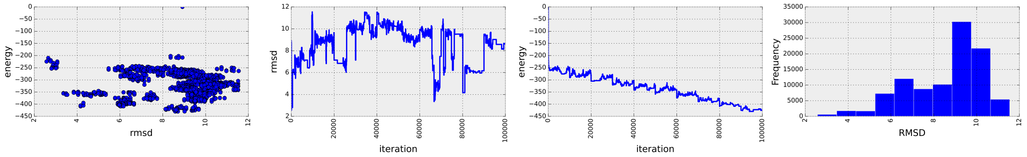

1KXK [70]

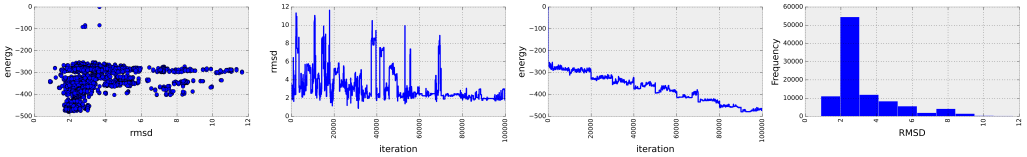

1Y26 [71]

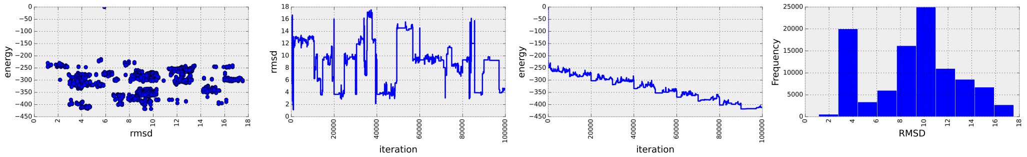

2TRA [73]

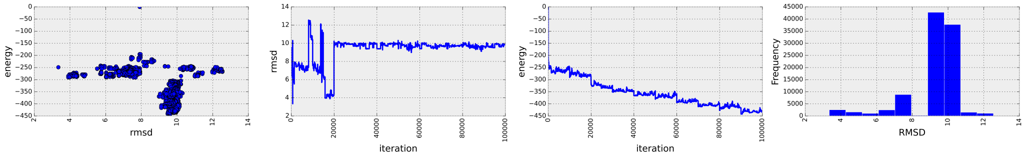

Continued on next page

2HOJ [78]

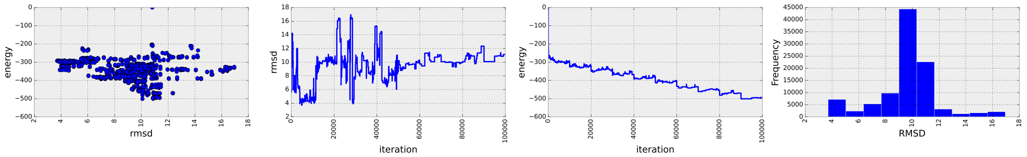

4P5J [83]

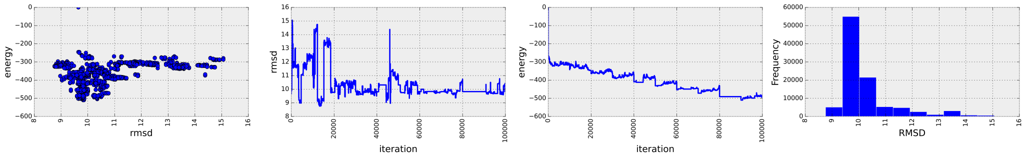

3T4B [83]

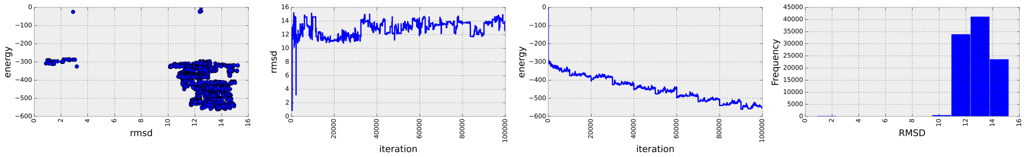

4FRG [84]

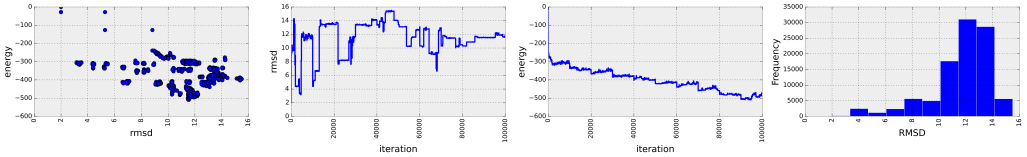

4LVZ [89]

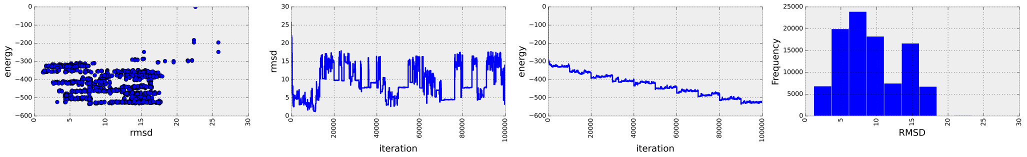

3GX5 [94]

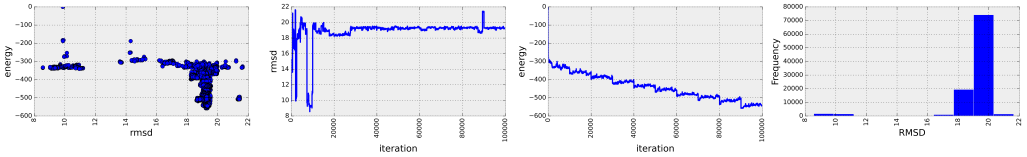

4L81 [96]

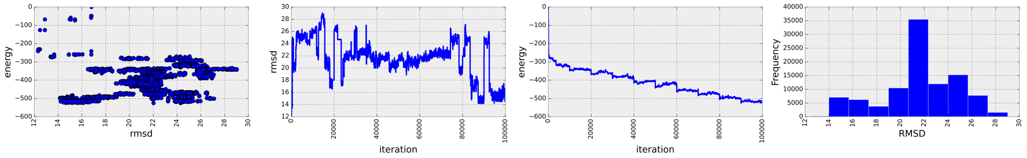

Continued on next page

Table A.3 – continued from previous page

2QBZ [153]

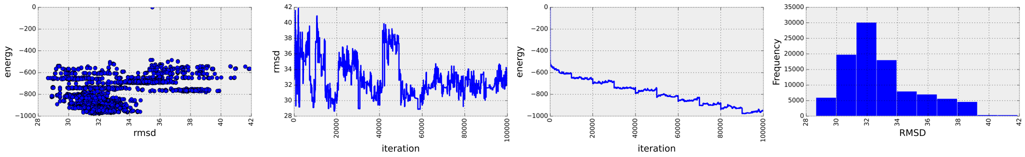

1U9S [155]

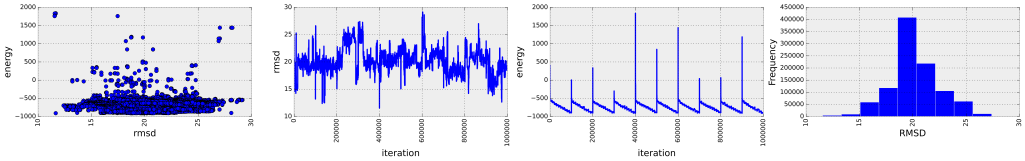

1GID [158]

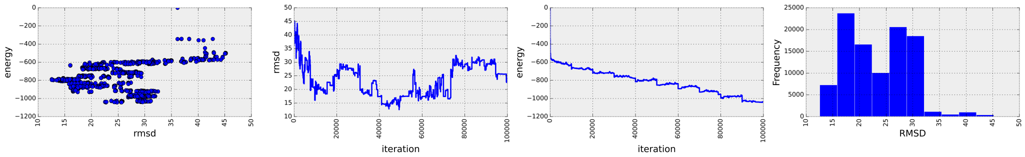

3D0U [161]

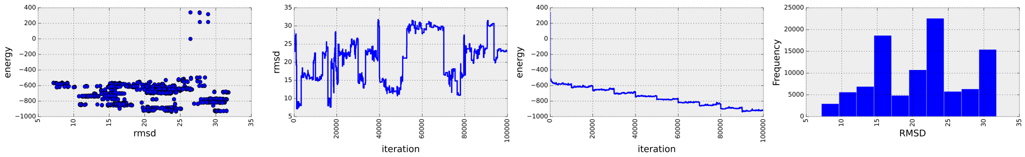

3DIR [172]

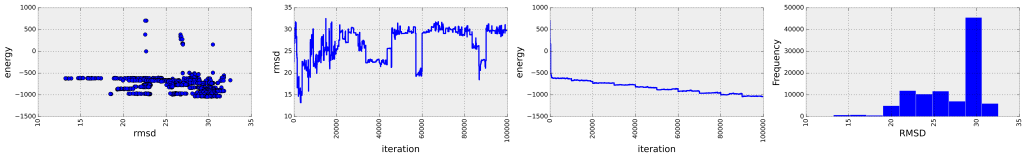

4P8Z [188]

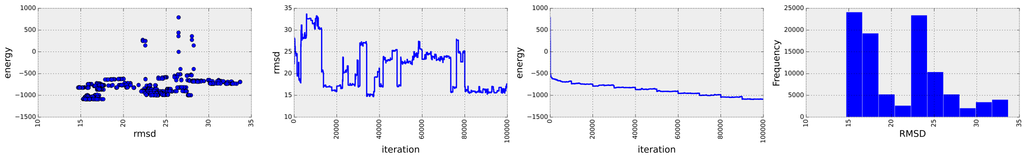

4P9R [189]

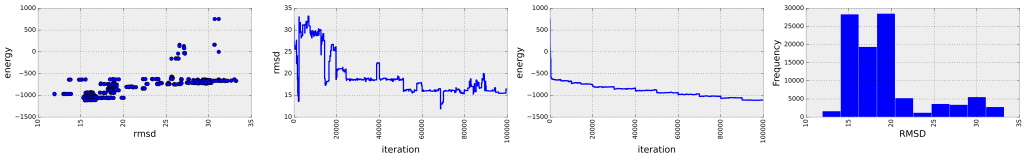

Continued on next page

Table A.3 – continued from previous page

4GMA [192]

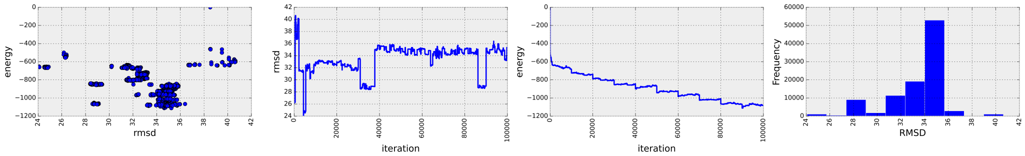

3DHS [215]

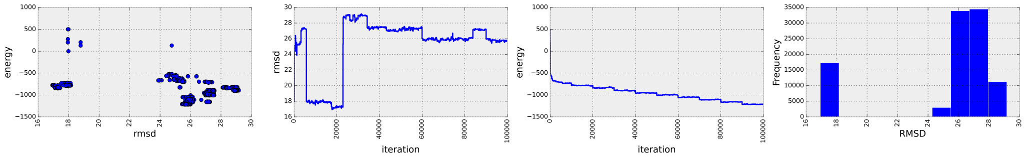

1X8W [247]

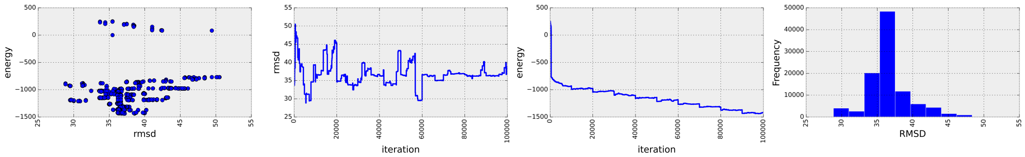

A.8. Running Time

The times listed in Table A.4 represent the entire time spent for each simulation. Due to the lack of sequence dependence, all fragment statistics used by Ernwin are precomputed so normal usage does not require their recalculation. FARNA, however, requires the creation of sub-models for each loop region, leading to the large running time listed in Table A.4. Such models could, in principle, be computed ahead of time, stored externally and assembled to form a complete structure which would make simulations using FARNA significantly faster.

PDB ID	Chain Length	Ernwin	NAST	RNAComposer	FARNA
3R4F	66	1475	319	16	5482
4PQV	68	1812	235	15	6141
1FIR	69	952	48	14	6422
1YFG	69	1034	262	14	6319
1KXK	70	2284	277	15	5592
1Y26	71	1775	48	13	6747
2TRA	73	1282	150	13	6444
3CW5	75	1915	316	16	*
2HOJ	78	3332	341	16	7381
4P5J	83	2004	225	17	8669
4FRG	84	2993	63	17	9227
4LVZ	89	1976	302	20	8047
3GX5	94	4469	171	20	10751
4L81	96	2982	277	21	10539
2QBZ	153	3529	536	31	14549
1U9S	155	9204	*	35	21692
1GID	158	3852	328	33	15047
4GXY	161	6171	*	34	*
3D0U	161	5395	354	36	16796
3DIR	172	3167	485	38	16832
4P8Z	188	3398	1530	39	19630
4P9R	189	3607	*	39	20118
4GMA	192	4122	1354	47	22965
3DHS	215	4279	446	52	30740
1X8W	247	6334	1194	71	28799
2A64	298	2687	*	72	*

Table A.4: The total time (in seconds) spent assembling structures. It should be noted that the results are a run of 10000 MCMC steps using Ernwin, 10000 MCMC steps for 10 structures using FARNA, 1000000 MCMC steps using NAST and obtaining 10 structures using the RNAComposer web interface. Asterisks denote a failure to obtain models using the given program. The times correspond to the samples and predictions presented in Figure 8.
